# The thymus and T-cell ontogeny in ballan wrasse (*Labrus bergylta*) is nutritionally modelled

**DOI:** 10.3389/fimmu.2023.1166785

**Published:** 2023-05-01

**Authors:** Angela Etayo, Kai K. Lie, Reidun M. Bjelland, Ivar Hordvik, Aina-Cathrine Øvergård, Øystein Sæle

**Affiliations:** ^1^ Feed and Nutrition group, Institute of Marine Research, Bergen, Norway; ^2^ Fish Health Group, Department of Biological Sciences, University of Bergen, Bergen, Norway; ^3^ Institute of Marine Research, Austevoll Research Station, Storebø, Norway

**Keywords:** adaptive immunity, lymphoid, thymocytes, larval ontogeny, early nutrition

## Abstract

Marine fish larvae often experience high mortality unrelated to predation during early life stages, and farmed ballan wrasse (*Labrus bergylta*) is no exception. Knowing when the adaptive immune system is developed and fully functional, and how nutrition may modulate these processes is therefore of importance to establish effective prophylactic measures and will also extend the relatively limited knowledge on the immune system in lower vertebrates. The thymus anlage of ballan wrasse was found to be histologically visible for the first time at larval stage 3 (20–30 days post hatch, dph) and becomes lymphoid at stage 5 (50–60 dph) correlating with an increase of T-cell marker transcripts. At this stage, a clear zonation into a *RAG1^+^* cortex and a *RAG1^-^ CD3ϵ^+^* medulla was distinguished, indicating that T-cell maturation processes in ballan wrasse are similar to other teleosts. The higher abundance of *CD4-1^+^* compared to *CD8β^+^* cells in the thymus together with the apparent lack of *CD8β^+^* cells in gill, gut, and pharynx, where *CD4-1^+^* cells were identified, indicates that helper T-cells have a more prominent role during larval development compared to cytotoxic T-cells. As ballan wrasse lacks a stomach but has an exceptionally high IgM expression in the hindgut, we hypothesize that helper T-cells are crucial for activation and recruitment of IgM^+^ B-cells and possibly other leukocytes to the gut during early development. Nutritional factors such as DHA/EPA, Zn and Se may lead to an earlier expression of certain T-cell markers as well as a larger size of the thymus, indicating an earlier onset of adaptive immunity. Including live feeds that supplies the larva with higher amounts of these nutrients can therefore be beneficial for ballan wrasse farming.

## 1 Introduction

Infections with the marine ectoparasitic copepod, the salmon louse (*Lepeophtheirus salmonis*), is a major problem for the salmon farming industry. In order to avoid heavy infestations of salmon, ballan wrasse (*Labrus bergylta*) is used as a cleaner fish for ectoparasite countermeasure. Ballan wrasse farming was initiated to decrease the fishing pressure on wild wrasse stocks, but as of now it is a relatively new industry with room for improvement. Efforts have been made to optimize feeding practices during early life stages (1), and the development of wrasse larvae has been described with focus on the ontogeny of the digestive system (1, 2). In more recent years, research on wrasse intestinal physiology and functionality has described some of the evolutionary traits of this stomach-less fish (3–5). However, wrasse farming still faces many challenges such as poor growth and a high mortality, especially during early life stages as in many other farmed marine teleost species (6, 7). The bacterial diseases Atypical *Aeromonas salmonicida* (aAs) and *vibrio anguillarum* are the primary challenge in farmed wrasse in sea pens and sporadic outbreaks have also occurred in hatcheries (8). It is believed that maternal transfer of defense molecules such as lectins and IgM to the oocytes can improve robustness at embryonic and larval stages until adaptive immunity (B-cells and T-cells) becomes functional (9, 10). After the appearance of B- and T-cells, long lasting memory is believed to be established, and the larva becomes better protected against pathogens and can be vaccinated. Understanding the ontogeny of the adaptive immune system, and specifically the appearance of functional lymphocytes, is therefore crucial for the development of efficient vaccination protocols.

T-cells are together with B-cells, the key cellular fraction of the adaptive immune system in vertebrates. Mammalian T-cells are characterized by having a T-cell receptor complex (TCR/CD3) that recognizes antigenic peptides on the surface of the major histocompatibility complex (MHC) molecules, known as MHC restriction. The majority of mammalian T-cells contain a TCR formed by the αβ heterodimer, whereas the TCR-γδ T-cell populations is relatively small (11). Moreover, there are two main subsets of T-cells distinguished by the expression of two coreceptors, CD4 and CD8. CD4^+^ T-cells can be regulatory cells (Treg) that are key in mucosal homeostasis and immune regulation, and helper cells (Th) that secrete cytokines stimulating other immune cells. CD8^+^ T-cells, the so-called cytotoxic cells (Tc), directly kill cells infected by pathogens such as viruses and intracellular bacteria (12). Teleost T-cells seem to resemble those in mammals. Several genes expressed in T-cells, such as *TCRαβ, TCRγδ, CD3* (ϵ*, γ*, and *δ*), *CD4* (*-1* and *-2*), and *CD8* (*α* and *β*) have been described in several teleosts as recently reviewed in Barraza et al. (13). Ballan wrasse *TCRα, TCRδ*, and *CD3ϵ* have also been characterized (14, 15).

The thymus is the major site for T-cell development, and thus a key organ in the immune system. The mammalian thymus is a bi-lobed organ divided into two zones: the cortex (outer zone) and the medulla (inner zone), each of them with well-defined microenvironments. Only around 5% of the cells entering the thymus will exit the thymus as mature T-cells expressing a functional TCR able to recognize peptides bound to MHC molecules while being tolerant to self-MHC/self-peptides (11). T-cell maturation is a complex process that is strictly regulated and requires constant contact of T-cell precursors with both stromal and thymic epithelial cells. The development of mammalian T-cells starts with the activation of the recombination-activating genes (*RAG1* and *RAG2*) involved in *TCR* locus rearrangement (11). Early lymphocytes or thymocytes are double negative (DN), not expressing CD4 nor CD8 (CD4^-^CD8^-^). DN thymocytes that are able to rearrange their TCR-β chain and express it on their surface together with CD3 chains, form a pre-TCR complex and will proliferate becoming double positive (DP) CD4^+^CD8*^+^* thymocytes. Cortical DP thymocytes that survive to positive selection, mature to single positive (SP; CD4^+^ or CD8^+^) T-lymphocytes and migrate to the medulla where further negative selection occurs. As a result, mature SP T-cells are RAG^-^, and can now enter circulation and migrate to secondary lymphoid organs where maturation of T cells is finalized upon antigen recognition (16, 17).

The teleost thymus is a paired organ located dorsally near the gill cavity (18, 19), enclosed by a capsule which consist on both epithelial cells and connective tissue. Epithelial cells are exclusively found in the region where the organ faces the gill cavity, whereas connective tissue appears surrounding the remaining portions of the thymus. Invagination of connective tissue in the thymus are called trabeculae and contain vascularized capillaries. Similar to higher vertebrates, the teleost thymus seems to be zoned into a cortex and medulla. Although the first description of a teleost thymus was in the eighties with the characterization of the rainbow trout (*Oncorhynchus mykiss*) thymus by Grace and Manning (20), it took some years before researchers started to address the molecular mechanisms regulating T-cell maturation in fish. *RAG* genes have been shown to be expressed in the early thymus of zebrafish (*Danio rerio*) (21), medaka (*Oryzias latipes*) (22), common carp (*Cyprinus carpio*) (23), Atlantic halibut (*Hippoglossus hippoglossus*) (24), and Atlantic salmon (*Salmo salar*) (25) among others, with a cortex restricted expression shown in species with a zoned thymus. DP thymocytes and both subsets of SP T-cells (CD4^+^ and CD8^+^ T-cells) were found in sea bass (*Dicentrarchus labrax*) (26), ginbuna crucian carp (Carassius *auratus* langsdorfii) (27), and rainbow trout thymus (28, 29). The expression of MHC class I and II in cortical thymic epithelial cells (cTECs) is crucial for positive selection of thymocytes during T-cell maturation (30). In accordance with this, MHC II^+^ cells have been observed in the outer zone of the thymus in sea bass (31), Atlantic salmon (32) and rainbow trout (33). Furthermore, a series of genes coding for molecules that are either T-cell markers such as *LCK* and the 70 kDa zeta-associated protein (*ZAP-70*), but also play relevant roles in T-cell maturation as do the c-c chemokine ligand 25a (*CCL25α*) and the c-c chemokine receptors 9 (*CCR9*), have been cloned in teleost fish (13, 22). Altogether, findings support the idea that T-cell maturation is to some degree evolutionary conserved between fish and mammals. However, teleosts are very diverse, and studies comprising the thymic structure, zonation and T-cell development are restricted to few species and yet, with certain contradictions (13, 18).

Malnutrition is known to cause atrophy of the developing thymus and massive death of CD4^+^CD8^+^ thymocytes (34, 35) in mammals. Interestingly, nutrition has an important role at initiating and regulating B- and T-cell lymphocyte development in humans (36, 37). It is then assumable that nutrition affects development of adaptive immunity in teleosts, but corresponding studies are lacking. A well-known inherent challenge in larvae requiring live feed is to provide good nutrition (38). The most common dietary practice for small marine larvae is to give enriched rotifers followed by enriched Artemia. Rotifers and artemia are prey animals that are easy to provide for the larvae, but offer suboptimal nutrition (39) and therefore, they are enriched with essential nutrients before being administered to larvae.

To understand the timing when functional lymphocytes appear in ballan wrasse larvae, we here present the histological and molecular ontogeny of the thymus and developing T-cells, as well as the potential of a barnacle nauplii start-feed diet to boost an earlier ontogeny of the adaptive immune system compared to larvae fed with enriched rotifers and artemia.

## 2 Materials and methods

### 2.1 Experimental design

**Figure 1:**
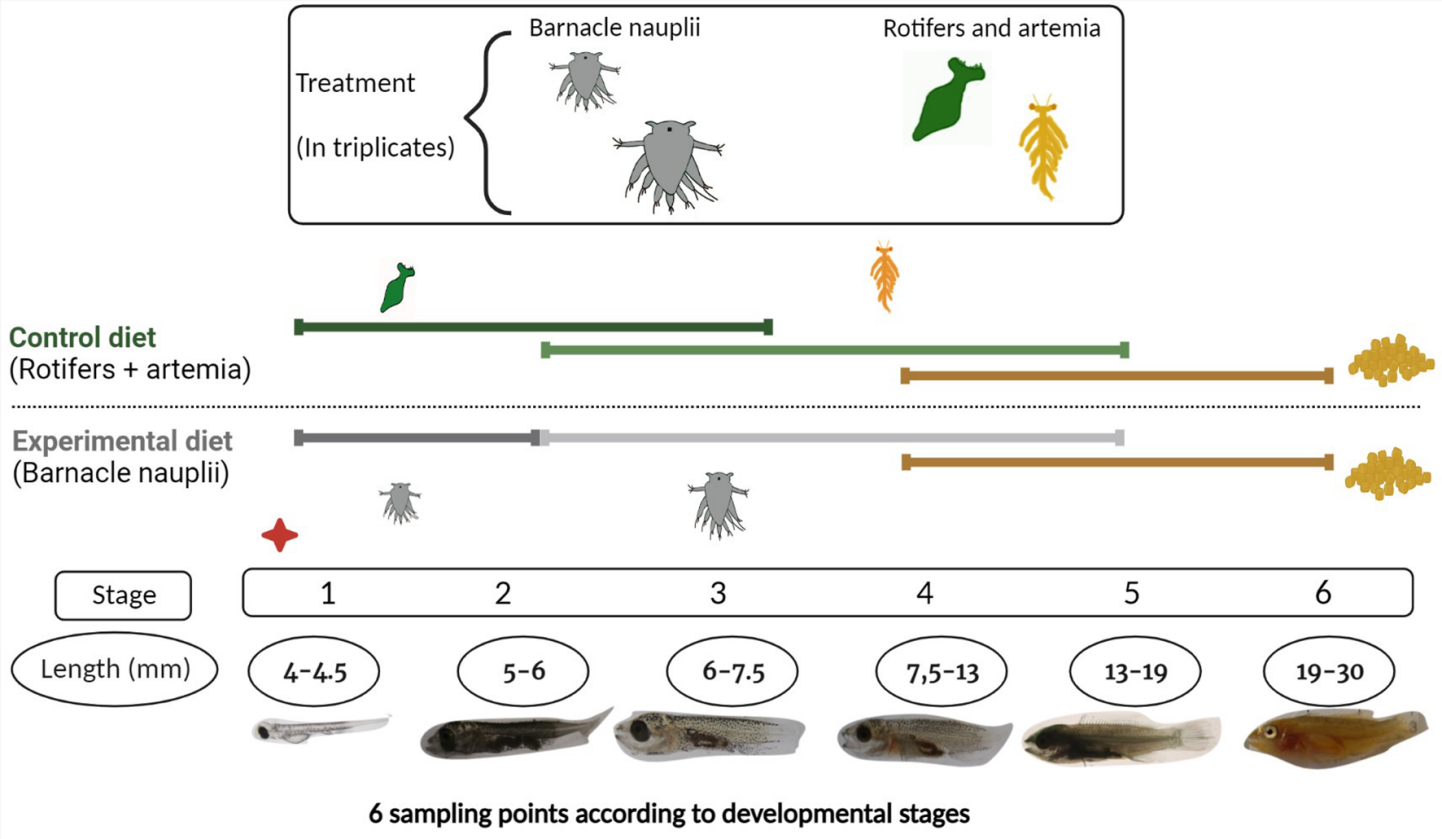
Experimental design. Two start-feed diets were given in triplicates: a control diet (rotifers and artemia), and an experimental diet, so-called barnacle diet (barnacle nauplii). The control and barnacle diet are represented in green and grey color respectively and colors are used henceforth for easier interpretation of results. Six sampling points were done according to developmental stages of wrasse larvae. After stage 6 they were considered juveniles. The red dot indicates the start of the feeding trial at 4 dph with rotifers and small barnacle correspondingly. Artemia and large barnacles were introduced at stage 2 and commercial feed (pellets) were introduced in a co-feeding regime at stage 4. Created with BioRender.com.

Brood stock ballan wrasse (30 females and 6 males) were kept in tanks at 8°C from October to the end of February of 2020 (2 weeks before spawning start). The temperature was then raised to 12°C during the spawning season and until September. Brood stock fish were fed to saturation every day. After spawning, eggs were placed in incubators with a capacity of 250 L at 12°C, water flow of 5 L/min and natural light until hatching. At 4 days post hatching (dph), 30 000 to 34 000 individuals were transferred into six different tanks and two different start-feed diets were given in triplicates. At this stage, most of the larvae had completely depleted their yolk-sac. The control diet consisted of rotifers enriched with algae (*Nannochloropsi*s and *Tetraselmis*) from Microalgae AS, Vigra, Norway, followed by artemia enriched with LARVIVA Multigrain (BioMar), cultivated, and enriched at the in-house facility at IMR, Austevoll, Bergen (Norway). The experimental diet was barnacle nauplii of two different sizes (small and large barnacle) from Planktonic company that were frozen in liquid nitrogen and revived before being added to the tanks. Detailed information regarding cultured conditions of rotifers, artemia and barnacles can be found in **Supplementary Data 1**. The larvae were kept in tanks with a capacity of 500 L at 15°C, with a starting water flow of 50 L/h that increased as the larvae grew, and a light regime of 24 hours (h). Commercial formulated feed (dry feed) was introduced at 40 dph in a co-feeding regime until 56 dph. After this time point, only commercial feed was supplied to all tanks. A total of six sampling points were set according to the six developmental stages of wrasse larvae that are based on the ontogeny of cranial ossification (2). The larval stages and the experimental feeding regime are summarized in **Figure 1**. At each sampling point a series of pooled larvae (3 to 15 larvae per pool) in replicates were collected from each tank, rinsed with distilled water, and immersed in RNA later at 4°C overnight and kept at -20°C until further use.

### 2.2 Nutrient analyses

Rotifers, artemia and both small and large barnacles were sampled in triplicates (n=3). Within each replicate, rotifers, artemia and barnacles were taken from their corresponding hatcheries the same day in the morning after feeding the larvae (approx. 10:30 am). Samples were passed through a sieve to concentrate the live prey and rinsed with distilled water to get rid of seawater. Samples were then aliquoted in different tubes for the different nutrient analyses and rapidly placed in dry ice and further stored at -80°C. One of the replicates was taken in the spring of 2020 and the other two replicates were taken in the spring of the following year. Analyses of proteins (aa composition), vitamins, pigments, and fatty acid composition was done on wet material. Analyses of minerals (ICP), total lipid and ash were done on dry material. References of the methods for nutrient analysis are in **Supplementary Table 1**.

### 2.3 RNA isolation and RNA-seq analyses

Two replicates of pooled larvae were collected from each tank (technical replicates) at each sampling point. The number of pooled larvae (per replicate and tank) varied from 15 individuals at stage 1, to 3 individuals at stage 6, sampling three biological replicates (tanks) for each time point (n=3). Pools of larvae were individually crushed in a mortar kept at –80°C. Fine powder was collected and used to isolate total RNA with QIAzol reagent^®^ (Invitrogen, Waltham, MA, United States) including DNase treatment (TURBO DNase, Ambion) according to the manufacturer’s protocol. RNA quality and integrity were assessed using a Nanodrop spectrophotometer (NanoDrop Technologies, Wilmington, DE,United States) and the 2100 Bioanalyzer (Agilent Technologies, Waldbronn, Germany). Total RNA samples were sent to Novogene Europe, Cambridge, UK, for sequencing using the Illumina NovaSeq 6000 platform for 150 bp paired end reads. CDNA libraries were prepared from individual samples and sequenced following manufacturer’s instructions and according to the Novogen pipeline (Novogene Europe, Cambridge, UK). Raw sequence reads were mapped against the ensemble wrasse gene build (Labrus_bergylta.BallGen_V1.104) using the Hisat2 mapper (40). Gene counting was conducted using feature counts v1.6.0 (41) as previously described (42). The count data was further normalized for differences in library size applying weighted trimmed mean expression ratios [trimmed mean of M values (TMM)] featured in the EdgeR package v 3.34 (43). Due TCR genes not being predicted in the wrasse ensemble gene build and the fragmented nature of the current wrasse assembly, especially for the immune genes with variable domains, a modified version of the original transcriptome (3) was made by extracting sequences related to *IgD, IgM, IgT, pIgR, TCRα* and *TCRδ* and replacing them with recently curated sequences (14, 44). To analyze the presence of *TCRα* and *TCRδ* in the different stages of wrasse, we conducted a re-mapping of all samples against the modified transcriptome using Salmon version 0.11.3 for mapping and quantification according to (45).

The raw data are available from the Sequence Read Archive (SRA) at the National Center for Biotechnology Information (NCBI) (Accession ID: SRX14748182). In this study, 18 genes of interest related to T cell development (*RAG1, RAG2, IKZF1, LCK, ZAP70, CD3δ, CD3ζ, CD3ϵ, CD4-1, CD4-2, CD8β, MHCII-α, MHCIIβ, CD74α, TCRα, TCRδ, CCR9β*, and *CCL25α*) were extracted from the RNA-seq data set and studied through larvae development using the Qlucore Omics Explorer v3.2.

### 2.4 Histology

For histological analyses of the thymus a total of 36 larvae across the six sampling points (n=3) were fixed and stored in Karnovsky fixative until further processing. Larvae in stage 3 to 6 were beheaded and the head was decalcified in EDTA 0.4 M, pH 7.2 for 2 to 7 days at 4°C. The solution was changed every other day. Larvae in stage 1 and 2 were used as whole and did not require decalcification. All larvae were then dehydrated through an ethanol gradient series up to 96% ethanol. Technovit 7100 kit (Kulzer GmbH) was used for resin embedding according to the manufacturer instructions. In short, dehydrated specimens were placed in the pre-infiltration solution (ethanol 96% and 50% Technovit 7100 basic solution 1:1) for 1.5 h followed by infiltration solution (hardener 1) overnight. The larvae and heads were orientated vertically with the mouth facing down in the mold and polymerization of the resin with hardener 2 solution was done in a desiccator for 24 h at room temperature (rt).

To localize and visualize the whole of the organ thymus, serial cross-sections of 2 µm were done using a Leica RM2165 microtome. Sections were collected from the cranial end, right at the back of the fish larvae’s eyes, until the thymus was not present in the sections. They were further stained with borax buffered toluidine blue.

### 2.5 Volumetric analyses

The volume of the thymus was investigated in a total of 18 larvae belonging to stage 4, 5, and 6 (3 larvae per diet and per stage) using the previous histological sections. Every fourth (for stage 4), every seventh (for stage 5), and every eleventh (for stage 6) sections were scanned using a semi-automatic scanner Nano Zoomer S60 (Hamamatsu, Japan) and visualized using NDP.view2 (Hamamatsu, Japan). Volumetric analyses were done by manually drawing a line around the thymus surface on selected slides, and the program further calculated the size of the marked area. The volume of the whole organ was then estimated as the thickness of each section times the total number of serial sections including the thymus.

### 2.6 Production and validation of antisera raised against wrasse CD3ϵ peptide

**Figure 2:**
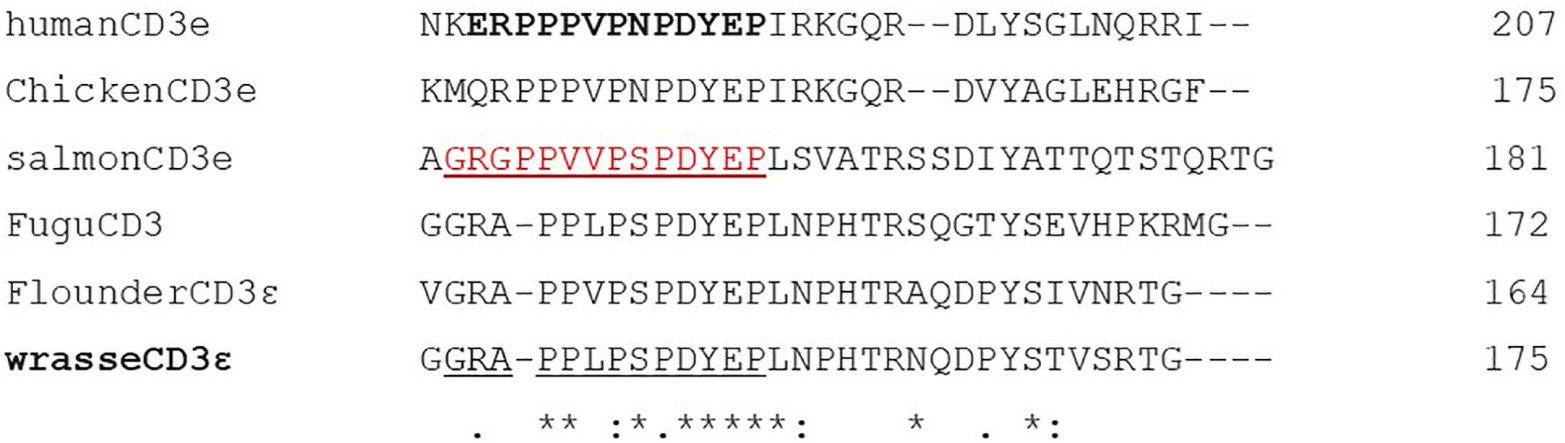
Alignment of the cytoplasmatic tail of wrasse CD3ϵ and corresponding sequences from human, chicken, salmon, fugu, and flounder. Residues identical in all sequences, highly conserved sequences, and conserved sequences are indicated by stars (*), colons (:), and periods (.) respectively. A commercial human antibody was raised against the peptide indicated in bold. In salmon, a successful antibody was raised against the peptide indicated in red. The corresponding sequence in wrasse is underlined and was used to raise an antibody (anti-wrasse CD3ϵ).

A polyclonal anti-wrasse CD3ϵ antibody was made as described in (46, 47). Wrasse CD3ϵ contains a cytoplasmatic peptide which is phylogenetically conserved among humans, higher vertebrates, and to a large extent, teleost fish (**Figure 2**). The corresponding peptide in ballan wrasse (GRAPPLPSPDYEP) was synthetically produced and used to immunize two rabbits according to the standard protocol of the producer. The resulting sera (anti-wrasse CD3ϵ) was subsequently affinity purified using the corresponding peptide (Davids Biotechnologie GmbH, Regensburg, Germany).

The anti-wrasse CD3ϵ was further validated by western blot analysis of different wrasse tissues. Wild ballan wrasse (700 - 900 g) were caught from fjords close to Bergen, Norway. They were anaesthetized with MS-222 (30 mg/ml) and sacrificed by a blow to the head. Thymus, head kidney, liver, muscle, spleen, gills and hindgut were excised and washed in cold PBS mixed with protease and phosphatase inhibitors (Pierce™). Tissues were homogenized in lysis buffer (4% SDS, 0.1M Tris-HCl pH 7.6) using a tissue disruptor and further sonicated using an ultrasonication rod (Q55 Sonicator, Qsonica, CT, USA) at 30% amplitude for 30 sec. Tissue lysates were centrifuged at 400 x *g* for 10 min at rt, the upper fraction incubated at 95°C for 5 min and further centrifuged at 15 000 x *g* for 10 min. The supernatant containing proteins was collected and quantified using the Bradford assay according to the manufacturer’s guidelines. Approximately 30 µg protein from each tissue were run on reducing, denaturing, 4–15% gradient gels. Western blotting was performed at 22 V and 1.3 A for 7 min at 22°C using a Trans-Blot Turbo System (Bio-Rad). To avoid unspecific binding of antibodies, the PVDF membrane was blocked for 30 min and incubated with rabbit anti-wrasse CD3ϵ (1:5000) for 2 h. The membrane was washed and incubated with HRP-conjugated anti-rabbit IgG (1:2000) for 1 h. The PVDF membrane was developed using ECL reagents (Pierce™ ECL Western Blotting Substrate).

### 2.7 Immunohistochemistry

Larva from stage 6 fixed in 4% paraformaldehyde (PFA) in phosphate buffered saline (PBS, pH 7.2) were paraffin-embedded and sectioned at 3 μm thickness using standard procedures. The slides were incubated on a heating plate at 37°C for 24 h, followed by 58°C for 1 h, before deparaffinization in xylene and hydration in graded ethanol dilutions to distilled water. Heat-induced epitope retrieval was performed at 80°C for 40 min in 0.01 M citrate buffer (pH 6) using a water bath. The slides were cooled down and subsequently washed in 0.01 M PBS (pH 7.3). Unspecific binding was prevented by incubating the tissues in 0.05 M tris-buffered saline (TBS, pH 7.6) with 2% BSA and 2% goat serum at rt for 1 h. Polyclonal anti-wrasse CD3ϵ primary antibody was diluted 1:100 in TBS with 1% BSA before application, and the slides were incubated for 1 h at rt. After rinsing with TBS with 0.05% tween (TBS-T), endogenous peroxidase was blocked by incubation in 1,5% Hydrogen peroxide solution (Merck KGaA, Darmstadt, Germany) at rt for 10 min, and several washes with TBS-T. The slides were then incubated with goat polymer-HRP anti-rabbit (abcam, Cat. No.: ab97051) 1:1000 for 45 min and developed with DAB substrate (Cell signaling, Cat. No.: 8059). Between each step, the slides were washed in TBS-T. Slides were further dipped in 0.01 M citrate buffer, pH 4.8, and then counterstained in Methyl green solution (Vector Laboratories, Cat. No.: H-3402) at 60°C for 20 min. Slides were quickly immersed in 0.01 M citrate buffer, pH 4.8, and blot dried before dehydration through 95% and 100% ethanol before mounting in non-aqueous VectaMount^®^ Mounting Medium (Vector Laboratories, Cat. No.: H-5000). As negative control, primary antibody was omitted from the procedure. The sections were imaged using a Leica DM 2500 LED with associated camera Leica DMC 6200. The software Leica Application Suite X was used.

### 2.8 *In situ* hybridization

*In situ* hybridization was performed on larvae in stage 5 and 6. To investigate development in more detail, the stages were divided into 2 substages according to larvae standard length (SL) and referred as early and late substage throughout the text. A total of 8 individual larvae were run in duplicates as followed; 2 larvae from early substage 5 (SL: 1,6), 2 larvae from late substage 5 (SL:1,8 cm), 2 larvae from early substage 6 (SL: 2,6), and 2 larvae from late substage 6 (SL: 3,5 cm) which are considered juveniles. The larvae were fixed in 4% PFA (pH 7.4) at rt for 24 to 32 h. Samples were then dehydrated, embedded in paraffin wax and 3 μm thick sections were made using standard procedures.

For *in situ* hybridization, RNA Scope 2.5 HD (Advanced Cell Diagnostics, Newark, CA, USA) probes for *RAG1, CD3ϵ, CD4-1* and *CD8β* were designed and produced by the manufacturer based on the provided sequences of ballan wrasse (**Table 1**). The *in situ* hybridization procedure was slightly modified from Løken et al. (48). In short, the paraffin-embedded tissue sections were mounted on positively charged glass slides (Superfrost, Mentzel), dried at 37°C for 48 h and further incubated at 60°C for 1 h. Subsequently, samples were de-paraffinized in 2 x 5 min xylene and 2 x 1 min 100% ethanol. Samples were treated for endogenous peroxidase blocking (10 min at rt), followed by target retrieval (15 min at 100°C), and protease digestion (30 min at 40°C) to allow permeabilization of cells. For probe hybridization, samples were incubated with the RNA scope probe for 2 h at 40°C, either as Duplex assays for simultaneous detection of two probes with the following combinations (*RAG1/CD3ϵ*, *CD3ϵ/CD4-1*, and *CD3ϵ/CD8β*), or as single assays targeting either *CD8β^+^* T- cells or *CD4-1^+^* T-cells. A series of hybridizations were performed using different incubation times according to the manufacturer´s instructions (49) to allow amplification of the signal. For signal detection, samples were then treated with chromogenic substrates bound to HRP (green color) and AP enzymes (red color) for 10 min each and subsequently stained with a 25% Gill´s hematoxylin solution for 30 sec. Samples were then dehydrated and mounted with non-aqueous VectaMount^®^ Mounting Medium (Vector Laboratories, Cat. No.: H-5000). The sections were scanned using a semi-automatic scanner Nano Zoomer S60 (Hamamatsu, Japan) and visualized using NDP.view2 (Hamamatsu, Japan).

**Table 1:** Probes used in *in situ* hybridization (*Labrus bergylta*).

	Probe	Accession no.	Target region (bp)	Catalogue no.
**Target**	RAG1	XM_020642835.2	1526-2425	1194681-C2
	CD4-1	XM_020649070.2	369-1437	1194661-C2
	CD8β	XM_020647965.2	131-1194	1194671-C2
	CD3ϵ	XM_020644379.2	103-1140	1194651-C1
**Control**	DapB (negative)	EF191515	414 - 862	310043
	EF-1a (positive)	XM_029279947.1	600-1592	1185171-C1

### 2.9 Statistical analyses

Statistical analyses were performed using Prism 9 (GraphPad Software Inc., CA, USA). The dry weight data violated the Shapiro-Wilk normality test and therefore, data were log-transformed before significance testing. Log-transformed data were normal distributed but presented unequal variances (F > F Critical one-tail using F-test for homogeneity of variances). As the main objective was to test the effect of the two start-feed diets on larvae growth at each developmental stage, the non-parametric Mann-Whitney test was selected as appropriate. A significant level of 0.05 was used.

For RNA-transcriptomic data, the aim was to see whether there was an effect of the start-feed diets on those genes related to T-cell development. For most of the selected genes, there were no transcript counts before stage 4. Therefore, statistics were only applied to the last three larvae stages (stages 4, 5, and 6). Data were log-transformed and are presented as means ± standard deviation (SD). F tests were performed to check for homogeneity of variances while normality was checked by the D’Agostino-Pearson test. Out of all the genes of interest, only *RAG1* and *ZAP70* were normally distributed and presented equal variances, and the parametric multiple t-test (Holm-Šídák t-test) was used for analyses of significances between the two start-feed diets within each stage. For *RAG2, CD3ϵ, TCRα*, and *IKZF1* normality or homogeneity in variance was not achieved, and the non-parametric Mann-Whitney test was performed. A significant level of 0.05 was used for all the tests.

Generalized Linear Model (glm) was applied to measure the effect of the start-feed diets on the volume of the thymus, considering the diets as categorical factor and larval myotome height (MH) (mm) as the continuous predictor. An ANOVA followed by Tukey’s multiple comparisons *post-hoc* test was used to compare the nutrient content among the life-preys (rotifers, artemia, small barnacle, and large barnacle) of the start-feed diets.

## 3 Results

### 3.1 Nutrient analyses

The complete nutrient analyses of the experimental start-feed diets (rotifers, artemia, small barnacle and large barnacle) are shown in **Supplementary Table 2**. An overview of the nutrients that are further discussed are shown in **Table 2**. Start-feed diets did not differ in the total amount of lipids nor in the amount of saturated fatty acids (SFA). Rotifers showed the lowest percent of monounsaturated fatty acids (MUFA) and the highest percent of n-3 docosahexaenoic acid (DHA). Both small and large barnacles showed significantly higher levels of n-3 eicosapentaenoic acid (EPA) and lower levels of the n-6 arachidonic acid (ARA) compared to rotifers and artemia (**Table 2**). As a result, the n-3/n-6 ratio was significantly higher in the barnacle diet.

**Table 2:** Overview of the selected nutritional analyses in rotifers, artemia, small barnacle (*Balanus crenatu*), and large barnacle (*Semibalanus balanoides)*.

	Rotifers	Artemia	Small barnacle	Large barnacle
Proximate composition (g/100g DW)				
Protein	51 ± 0^a^	36 ± 9^b^	42 ± 17^abc^	52 ± 22^ac^
Lipid	16 ± 4	13 ± 6	8 ± 4	9 ± 2
Ash	36 ± 11	23 ± 1	30 ± 18	23 ± 6
Dry weight (g/100g WW)	6 ± 4	9	22 ± 13	16 ± 5
Fatty acids (% of TFA)				
ΣSFA	22 ± 5	22	22	19
20:5n-3 EPA	7 ± 1^a^	9^a^	28^b^	32 ± 4^b^
22:6n-3 DHA	34 ± 6^a^	11 ± 2^b^	15^b^	19 ± 2^b^
20:4n-6 ARA	2^a^	3^b^	1^c^	1^c^
n-3/n-6	2^a^	2^a^	26 ± 2^b^	21 ± 7^b^
Micro-mineral composition (µg kg^-1^ DW)				
V	5 ± 1^a^	34 ± 47^ab^	88 ± 5^b^	26 ± 12^ab^
Mn	98 ± 29^a^	313 ± 429^a^	1170 ± 142^b^	159 ± 84^ac^
Co	2 ± 1^a^	7 ± 9^ab^	16 ± 1^b^	6 ± 4^ab^
Zn	203 ± 85^a^	2220 ± 2536^ab^	4343 ± 667^b^	5825 ± 843^b^
Se	3 ± 1^a^	10 ± 7^ab^	19 ± 3^b^	15 ± 2^b^
Jod	58 ± 26^a^	244 ± 300^a^	2819 ± 523^b^	736 ± 154^c^
Pigments (µg kg^-1^ DW)				
Astaxanthin	1374 ± 912^a^	41 ± 2 ^ab^	28 ± 20^b^	60 ± 10 ^ab^
Canthaxanthin	tr	tr	4*	7*
Non-essential amino acids (µg/Kg dw)				
Protein-bound amino acids (PAA)				
Proline*	676 ± 92	195 ± 1	188 ± 86	226 ± 10
Free amino acids (FAA)				
Proline*	52 ± 7	42 ± 2	35 ± 15	67 ± 4
Taurine*	3 ± 1	35	17 ± 7	25 ± 2

Values are relative to dry weight (DW) and are given as mean ± SD when value is >1. The number of replicates is 3 (N=3) unless otherwise specified by *(N=2). ANOVA test was applied only when N=3 and significances are indicated by letters.

Iodine was the only mineral that was significantly higher in both small and large barnacle diets compared to rotifers and artemia. Ash content was similar in all start-feed diets. The rest of the investigated minerals varied more between the four start-feed diets. Small barnacle was significantly higher in V, Mn, Co, Zn, As, Se, and Ca compared to rotifers (**Table 2**). As was found to be higher in large barnacle compared to artemia whereas artemia was higher in Na and Mg (**Supplementary Table 2**). Variation in nutrient content from batch to batch was especially high in artemia as batches were unfortunately taken in different years (same season). Rotifers and artemia were richer in all investigated vitamins compared to barnacles. Barnacles were devoid of vitamin D_3_, and Vitamin A was only found in trace levels in all diets. Enriched rotifers presented high levels of astaxanthin compared to artemia and barnacles whereas canthaxanthin was only detected in small and large barnacles at low levels (**Table 2**).

The concentration of both protein-bound amino acids (PAA) and free amino acids (FAA) was highest in rotifers and lowest in barnacles except for proline in its free form (FAA) that was highest in large barnacle, and the free amino acid taurine that was lowest in rotifers and highest in artemia (**Table 2**). The amino acid profile was similar among the start-feed diets where lysine, aspartic acid, and glutamic acid was the most abundant in the PAA fraction followed by leucine in rotifers and artemia, and glycine and proline in small and large barnacles respectively (**Supplementary Table 2**). The profile for FAA was similar between rotifers and artemia being arginine, lysine, and glutamic acid the most abundant amino acids. Differently, the FAA fraction of barnacle diets were rich in proline and alanine followed by glycine in small barnacles and taurine in large barnacles (**Supplementary Table 2**).

### 3.2 Growth and effect of start-feed diets during larvae ontogeny

**Figure 3:**
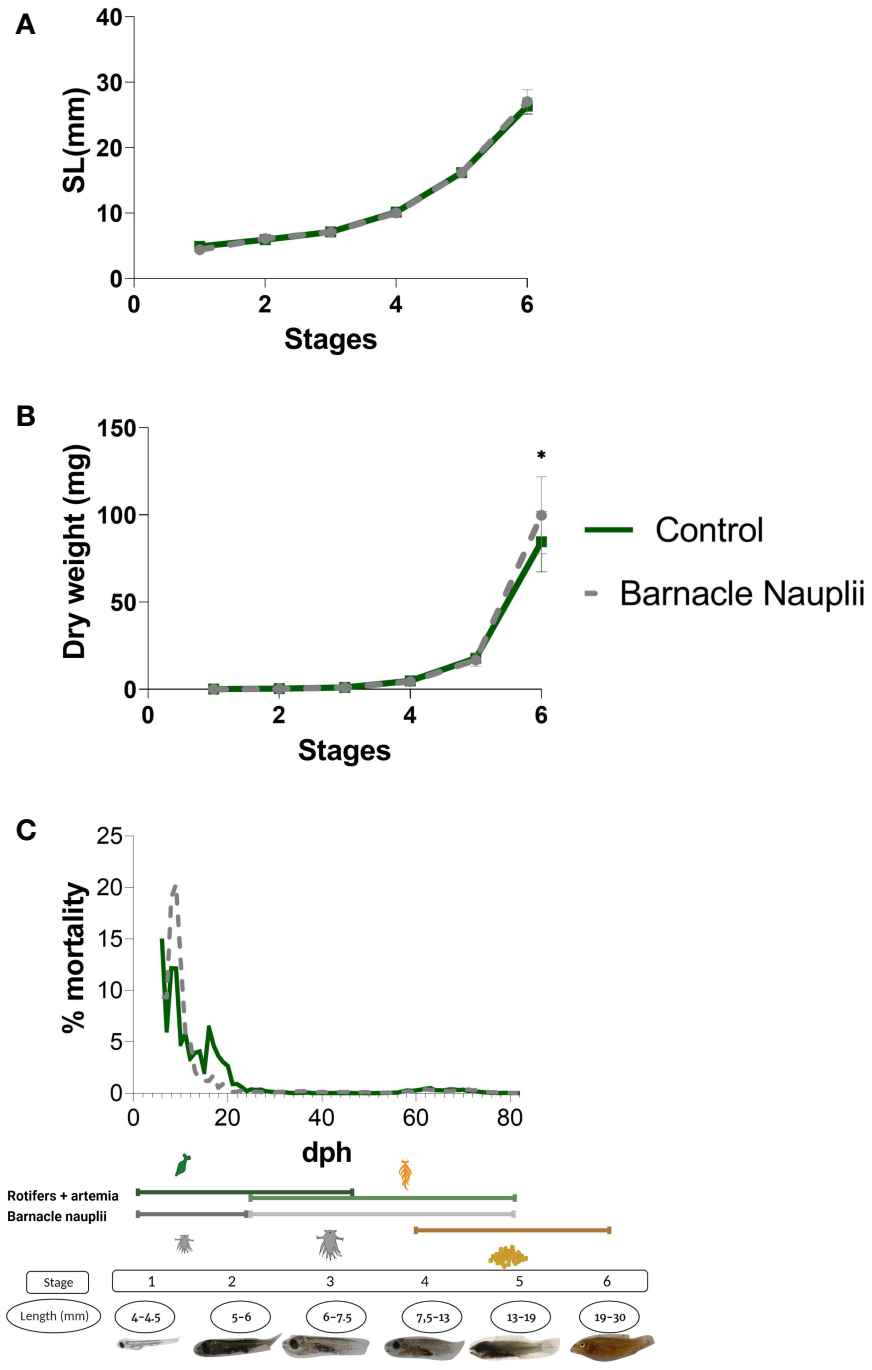
Figure 3. The dietary effect on growth performance and larvae mortality through ontogeny. **(A)** Larvae standard length (SL). **(B)** Larvae dry weight (DW) which was significantly higher in the barnacle nauplii diet in the last developmental stage (Mann-Whitney test, p value= 0.0287, indicated by *). **(C)** Mortality expressed in percentage. Stages and the feeding regime is included in the figure for eassier interpretation.

The two start-feed diets did trigger a significanlty higher dry weight (DW) of those larvae fed barnacle nauplii at stage 6 (Mann-Whitney; p value= 0.0287) (**Figure 3B**). Mortality peaked in larvae fed barnacle nauplii shortly after first feeding but decreased fastly to a mortality rate lower than the control diet group (rotifers and artemia) (**Figure 3C**). Mortality during weaning was similar between the two groups. Data corresponding to the standard length (SL) of sampled larvae (**Figure 3A**) is a confirmation of the successful execution of larvae collection at each given stage.

### 3.3 Transcription of T-cell markers and thymus-associated genes

**Figure 4:**
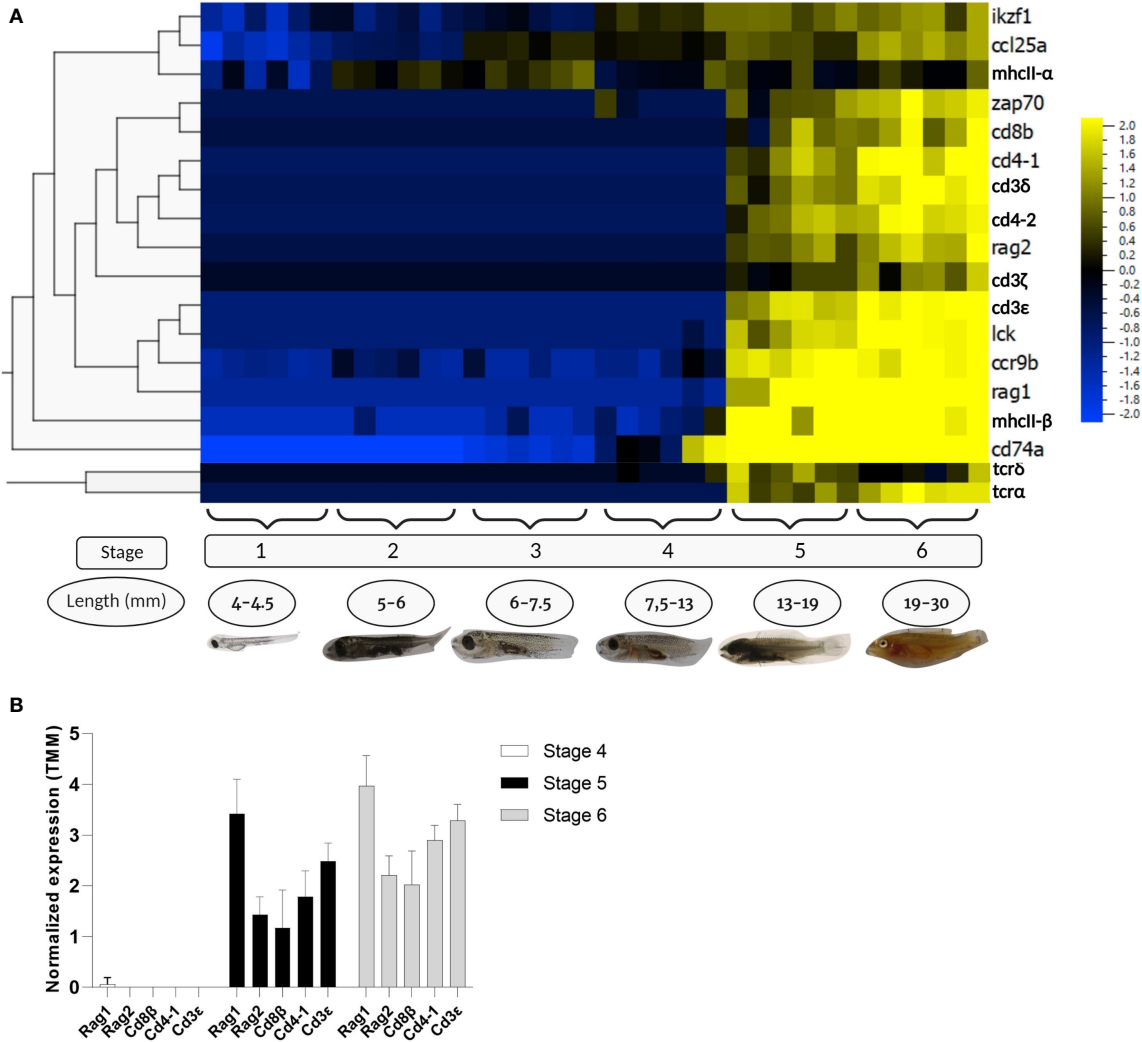
RNA transcriptomic data from different stages of wrasse larvae. Data were logarithm converted, normalized for differences in library size applying weighted trimmed mean expression ratios [trimmed mean of M values (TMM)], and expressed as mean = 0. **(A)** Heat map of RNA transcripts showing hierarchical clustering of the selected T-cell markers and non-lymphocytic thymus cells markers in the developmental stages of ballan wrasse. The figure was generated using Qlucore omics explorer software. **(B)** RNA transcripts of T-cell markers corresponding to *RAG1*, *RAG2, CD8β, CD4-1*, and *CD3ϵ* from larvae at stage 4, 5, and 6. Created with BioRender.com.

RNAseq data of wrasse larvae fed the control diet was analysed to study the starting point of T-cell development during ontogeny. In the present work, 18 genes corresponding to T-cell markers (*RAG1, RAG2, IKZF1, LCK, ZAP70, CD3δ, CD3ϵ, CD4-1, CD4-2, CD8β, TCRα, TCRδ*) and markers for thymic epithelial cells (*MHCII-α, MHCIIβ, CD74α, CCL25α*, and *CCR9β*) were examined (**Figure 4A**). Allthogether, the transcriptomic data indicated that T-cell maturation processes started at or just prior to stage 5 of wrasse development. The recombination-activating genes *RAG1* and *RAG2* were found to be upregulated from stage 5, though *RAG2* displayed a lower transcript level (**Figure 4B**) (the ratio *RAG1/RAG2* is 2.4 in stage 5, and 1.8 in stage 6). Expression of the T-cell markers *ZAP70*, the three CD3 chains (*CD3δ, CD3ζ, CD3ϵ*), *CD4-1, CD4-2, CD8β*, and *TCRα* also appeared at stage 5 but with fewer reads compared to *RAG1*, while *LCK* was the only gene displaying a similar level of expression as *RAG1* from stage 5 and onwards. Interestingly, only a small increase in the transcript level of the *TCRδ* chain was observed at stage 5, declining again at stage 6. On the opposite, transcripts of genes expressed in cortical and medullar epithelial cells important for thymus homing (*CCL25α*) and selection processes (*MHCIIα, MHCIIβ, CD74α* and *CCR9β*) were observed before stage 5, prior to the induction of *RAG1* and T-cell specific markers (**Figure 4A**). Similarly, the ikaros (*IKZF1*) transcription factor which is critial for early T-cell development appeared before stage 5.

### 3.4 Histology

**Figure 5:**
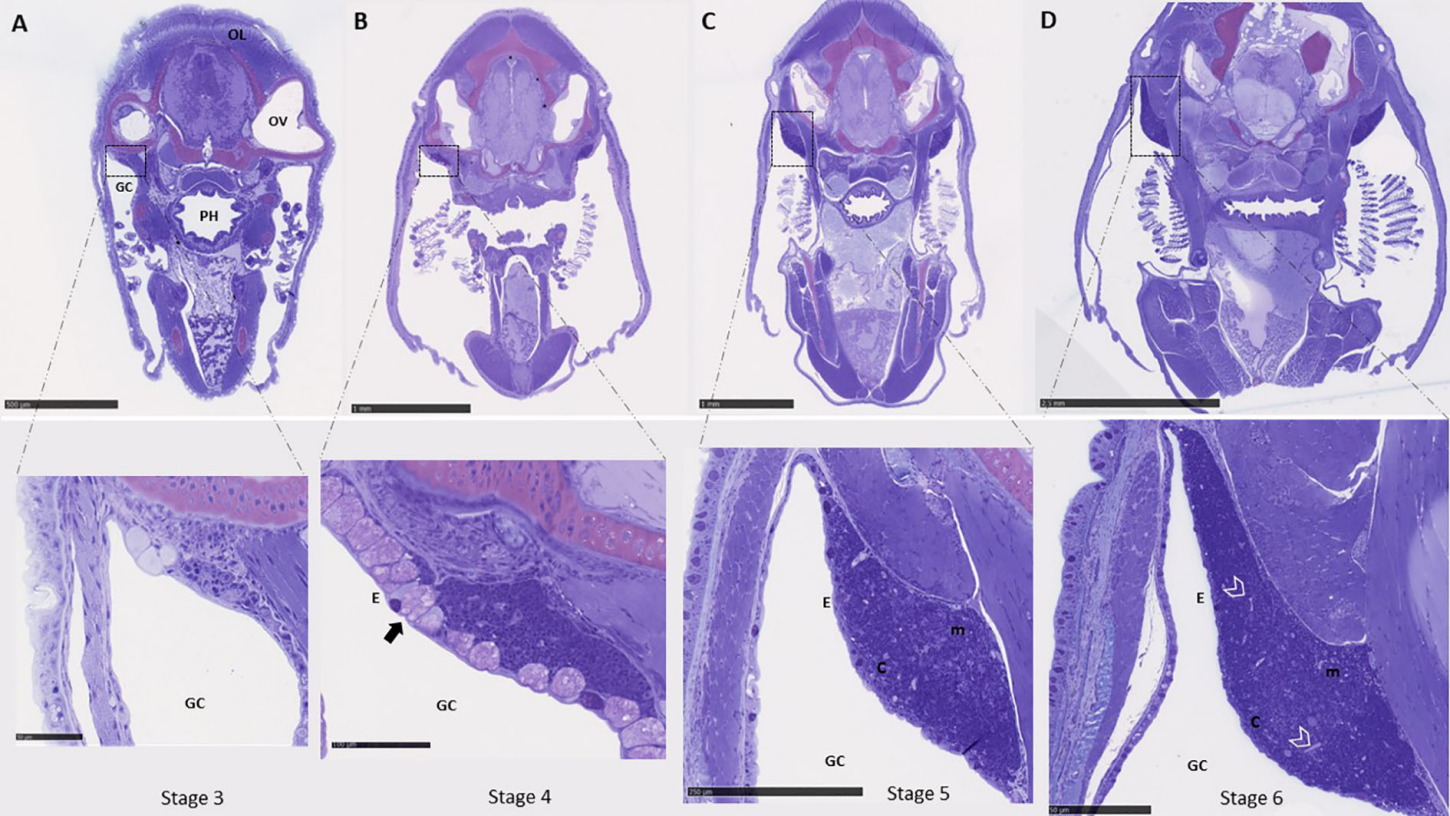
Histological examination of the thymus ontogeny in ballan wrasse. **(A-D)** show cross-sections of the whole head where the paired thymus can be observed dorsally in the opercular cavity. The left-side thymus is shown in higher magnification. **(A)** Correspond to larvae in stage 3 (SL: 6-7,5 mm). **(B)** larvae in stage 4 (SL: 7,5-13 mm). Mucous-like cells within the epithelium are indicated by an arrow. **(C)** larvae in stage 5 (13-19 mm), and **(D)** larvae in stage 6 (19 to 30 mm). Blood vessels are delimited by arrow heads. OL, Optic lobes; OV, Optic vesicle; PH, Pharynx; GC, Gill cavity; E, Epithelium; c, cortex; and m, medulla. Scales bars are as follows: **(A)** above 500 μm and below 50 μm, **(B)** above 1mm and below 100 μm, **(C)** above 1 mm and below 250 μm, and **(D)** above 2.5 mm and below 50 μm.

Based on the mRNA expression data, morphological studies of the thymus ontogeny were only done from stage 3 and onwards, which correspond to the stage prior to where the first transcripts were found to be slightly elevated in whole larvae. The first sign of the thymus anlage in ballan wrasse larvae was observed dorsally in the opercular cavity at stage 3 (**Figure 5A**), where distinct large undifferentiated cells and few thymocyte-like cells with high nucleus to cytoplasm ratio were detected. At stage 4, the thymus as an organ became morphologically distinguished and small cells with the characteristic morphology of thymocytes were more abundant. A high density of mucous-like cells was observed in the epithelium delimiting the thymus at stage 4 (arrow in **Figure 5B**) which corresponds to the start point of formulated feed, whereas none were observed at older stages. The thymus of larvae fed barnacle nauplii also showed abundant mucous-like cells in the epithelium exclusively at stage 4 (data not shown). The thymus became more prominent and a weak demarcation into cortex- and medulla- like zones was observed at stage 6 (**Figure 5D**), with a darkly stained cortex due to the higher density of thymocytes, and a paler stained medulla less densely packed with thymocytes. At stage 5 and 6, blood vessels were found to be more visible in the thymus parenchyma (**Figures 5C, D**). No apparent differences were observed in the morphology of the thymus between the two start-diet groups.

### 3.5 Validation of anti-wrasse cCD3e antibodies and immunohistochemistry of the thymus

The anti-wrasse CD3ϵ was found to react with a protein of the expected molecular mass of CD3ϵ in thymus (theoretical peptide weight 19,62 KDa), whereas no reactivity was observed in muscle used as control, gills, nor spleen (**Supplementary Figure 1**). A weak cross-reactivity was, however, detected in gills at approximately 75 kDa. The polyclonal anti-wrasse CD3ϵ antibody was used to identify CD3ϵ^+^ cells in the thymus of ballan wrasse larvae (**Supplementary Figure 1**). Cross reaction of the antibody was observed in neural tissue (especially in the ganglion) and epithelial cells, and therefore a monoclonal anti-wrasse CD3ϵ antibody is needed to be developed to avoid background staining.

### 3.6 *In situ* hybridization

**Figure 6:**
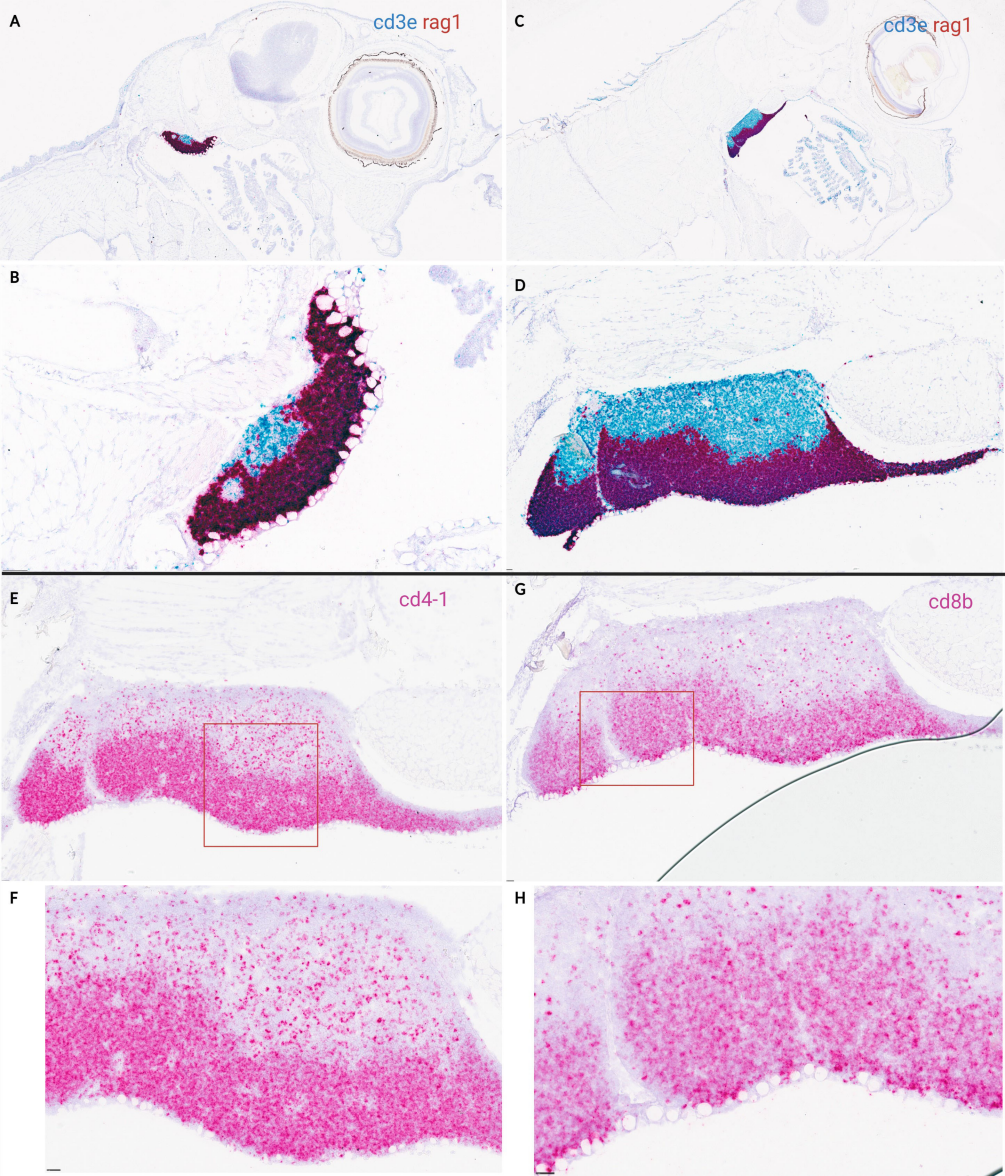
*In situ* hybridization on lateral sections of ballan wrasse thymus in larvae at stage 5 and juveniles. **(A-D)** shows duplex assays using RAG1(red) and CD3ϵ (blue) RNA scope probes**. (E-H)** shows *in situ* hybridization (single assay) on serial sections of juveniles’ thymus using either CD4-1probes **(E-F)** or CD8β **(G-H)** probes. **(A)** Larvae corresponding to early substage 5 (SL: 1,6 cm). **(B)** Higher magnification of **(A)**, **(C)** Juvenile (SL: 3,5 cm). **(D)** Higher magnification of **(C)**, **(E, G)** are juveniles (SL: 3,5 cm), and the area delimited by a red box is shown in higher magnification in **(F, H)** correspondingly. Scale bars: **(B)** 50 µm, and **(D-H)** 25 µm.

**Figure 7:**
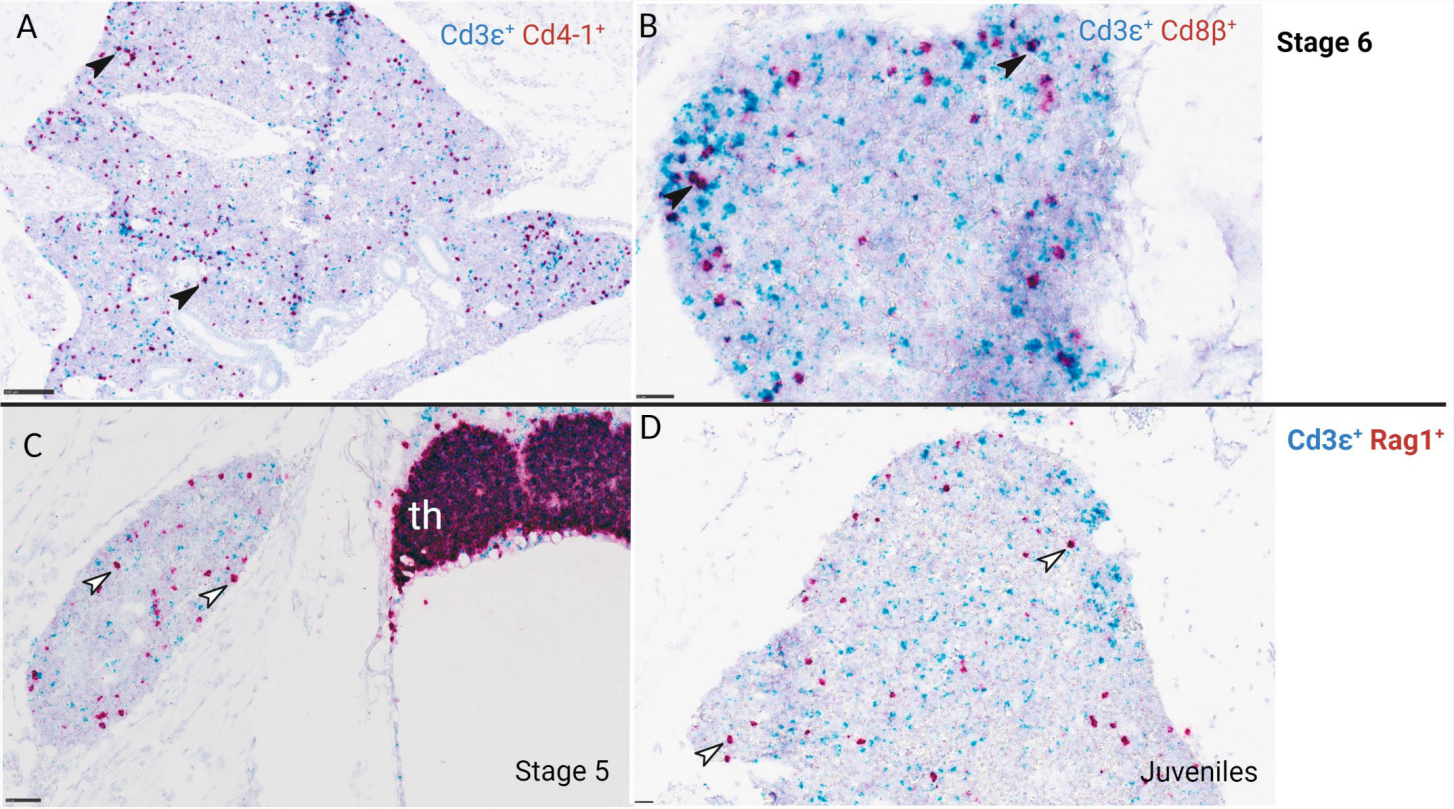
RNA scope *in situ* hybridization of ballan wrasse head kidney from larvae at stage 5, 6, and juveniles. **(A)** *CD3ϵ^+^* (blue) and *CD4-1^+^* (red) cells, larva at stage 6. **(B)** *CD3ϵ^+^* (blue) and *CD8β^+^* (red) cells, larva at stage 6. Black arrows indicate double stained lymphocytes that are either *CD3ϵ^+^ CD4-1^+^* T-cells or *CD3ϵ^+^ CD8β^+^* T-cells. **(C, D)** show *CD3ϵ^+^* (blue) and *RAG1^+^* (red) cells of larvae at stage 5 and juveniles correspondingly. White arrows indicate *RAG1^+^* cells in the head kidney. Th, thymus. Scale bars: **(A)** 50 µm, **(B)** 50 µm, **(C)** 100 µm, **(D)** 25 µm.

Based on transcriptomic data, *in situ* hybridization on lateral and cross-sections of ballan wrasse larvae was done on stage 5, 6, and juvenile fish, coinciding with the upregulation of transcripts corresponding to T-cell specific markers. *In situ* hybridization shows a clear demarcation between cortex and medulla in the thymus of ballan wrasse larvae (**Figure 6**). *RAG1* signal was prominent in the cortex at stage 5 but very scattered in the medulla whereas *CD3ϵ* was detected in the whole organ being clearly visible in the medulla (**Figures 6A, B**). The same pattern was observed in juveniles (SL > 3,5 cm) (**Figures 6C, D**). In accordance with this, *CD4-1^+^* and *CD8β^+^* cells were more abundant in the cortex than in the medulla (**Figures 6E–H**), probably staining DP and SP T-cell populations in the cortex and medulla, respectively. CD4-1^+^ cells were found to be more abundant compared to *CD8β^+^* in the thymus of investigated larvae (**Figures 6E–H**). Scattered *RAG1^+^* cells and *CD3ϵ^+^* cells were also observed in the head kidney of investigated larvae from stage 5, and onwards but to a much lesser extent compared to the thymus (**Figure 7**). *In situ* hybridization using the negative probe (*DapB*) did not give any signal.

**Figure 8:**
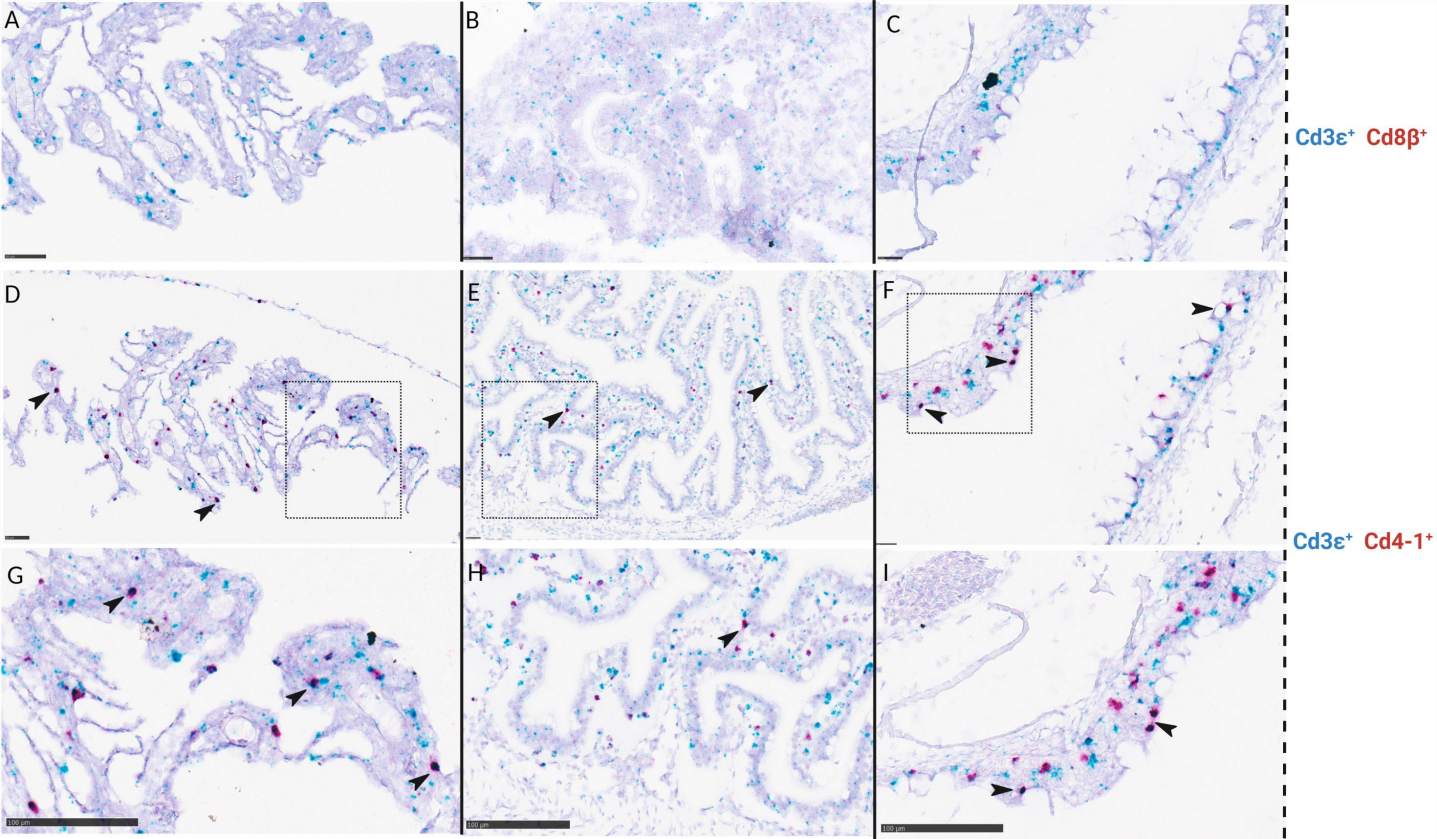
*In situ* hybridization of ballan wrasse juveniles (SL> 3,5 cm) shows differences in the amount of *CD4-1^+^* cells compared to *CD8β^+^* cells on serial sections of gills **(A, D,** and **G)**, gut **(B, E,** and **H)**, and pharynx **(C, F,** and **I)**. **(A-C)** are duplex assays using CD3ϵ (blue) and CD8β (red) RNA scope probes. **(D-F)** are duplex assays using CD3ϵ (blue) and CD4-1 (red) RNA scope probes. Areas delimited by a box in **(D-F)** are shown in higher magnification in **(G-I)** respectively. Arrows indicate putative *CD3ϵ^+^ CD4-1^+^* lymphocytes. Scale bars: **(A)** 50 µm, **(B)** 25 µm, **(C)** 25 µm, **(D)** 50 µm, **(E)** 25 µm, **(F)** 25 µm, **(G-I)** 100 µm.

Localization of *CD4-1^+^, CD8β^+^, CD3ϵ^+^*, and *RAG1^+^* cells were also analyzed in serial sections of gill and gut in a total of 8 larvae corresponding to stage 5, 6, and juveniles. *CD4-1^+^* cells were not observed in gill nor in gut at the early substage 5 (SL: 1,6cm) and very few were seen at late substage 5 (SL: 1,8 cm) (data not shown). The number of *CD4-1^+^* cells and putative helper T-cells (*CD4-1^+^ CD3ϵ^+^*) increased in gill and gut throughout stage 6 and juveniles (**Figures 8D–I**). *CD8β^+^* cells were not found in gill, gut, and pharynx of juveniles (**Figures 8A–C**). *CD8β^+^* and *CD4-1^+^* cells were observed in the head kidney of larvae at stage 6 (**Figure 7**).

### 3.7 Differential expression of RNA-transcripts

**Figure 9:**
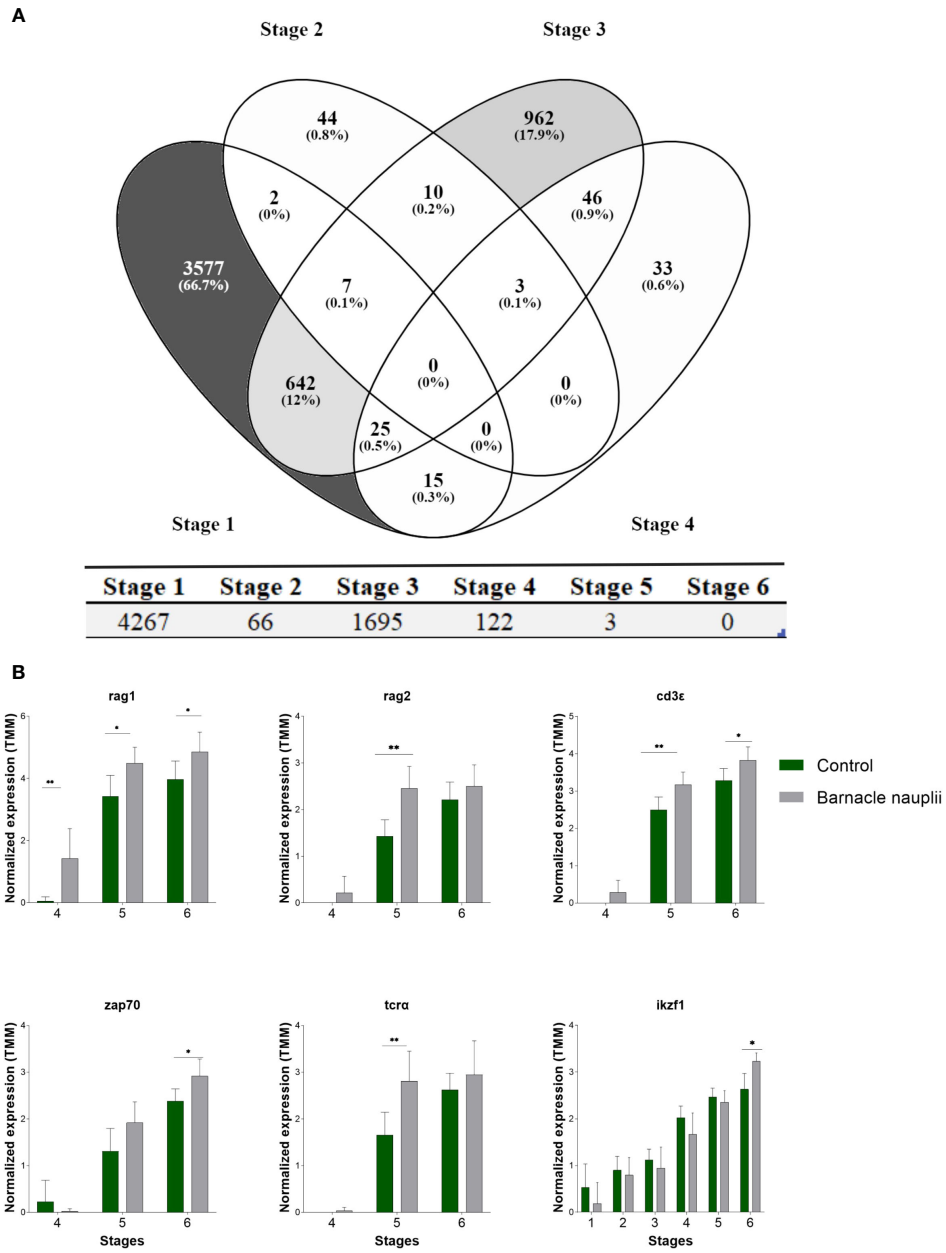
Effect of the start-feed diets on the transcriptome of ballan wrasse larvae at different developmental stages. **(A)** Venn diagram showing differentially expressed genes (q <0.05) in the first four larval stages triggered by diets (above panel). The total number of differentially expressed genes within each developmental stage (stage 1 to 6) is displayed below. **(B)** T-cell markers that were significantly affected by diets. Data correspond to transcription levels that were logarithm converted, normalized for differences in library size applying weighted trimmed mean expression ratios (trimmed mean of M values (TMM)), and presented as mean = 0, ± SD (n=3). When P ≤ 0.05 significances are represented by * and when P ≤ 0.01 significances are represented by **.

The two start-feed diets triggered large differences in the transcriptome within stages 1-4 whereas few genes were significanlty affected by diets (q< 0.05) in stage 5 and none in stage 6 (**Figure 9A**).

Among the selected T-cell markers in this study, the barnacle nauplii diet triggered earlier expression of the recombination-activating genes *RAG1* and *RAG2* (**Figure 9B**). Significances were as follow; *RAG1* (Holm-Šídák; p values of 0.01, 0.01, and 0.03 in stage 3, stage 4 and stage 5 correspondingly. *RAG2* (Mann-Whitney; p values of 0.01 in stage 5). Accordingly, *TCRα, CD3ϵ, ZAP70*, and ikaros (*IKZF1*) transcripts were significantly more abundant in the larvae fed barnacle nauplii (**Figure 9B**). Significances were as follow; TCRα (Mann-Whitney; p values of 0.02 in stage 5). CD3ϵ (Mann-Whitney; p values of 0.03 and 0.03 in stage 5 and stage 6 correspondingly). *ZAP70* (Holm-Šídák; p values of 0.04 in stage 6). *IKZF1* (Mann-Whitney; p value of 0.01 in stage 6). Only p values < 0.05 are hereby presented.

### 3.8 Thymus volume

**Figure 10:**
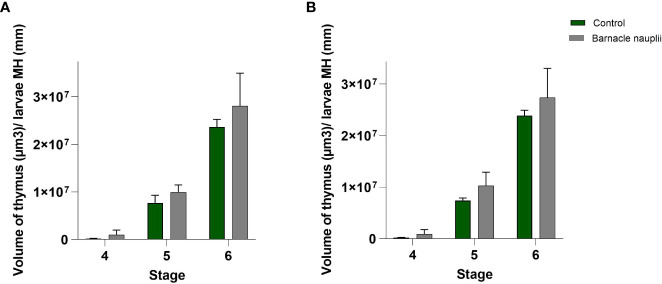
Volumetric analysis of the wrasse larvae thymus (n=3). **(A)** Left-side thymus and **(B)** Right-side thymus. MH: Myotome height. The barnacle nauplii diet triggered larger volume of the thymus (glm; p value=0.0008).

The volume of the thymus was measured in 3 larvae at each of the developmental stages 4, 5 and 6. The barnacle nauplii diet resulted in a significantly larger volume of both right and left side thymus (glm; p value = 0.0008) (**Figure 10**).

## 4 Discussion

The study describes the timing of thymus and T-cell development in ballan wrasse, a process that seems to be similar to that in other teleosts (50–53). The first histological observation of the wrasse thymus anlage was at stage 3 with few thymocyte-like cells which increased considerably in numbers at stage 4. During these stages (stage 3 and 4), mRNA transcripts of T-cell specific markers were absent indicating that T-cell maturation had not yet started. Although the development of other lymphoid organs has not been addressed in ballan wrasse, the thymus appeared at the same time as the gut starts rotating and acquire the characteristic intestinal loop of this a-gastric species (2) which is an important morphological trait of the digestive system. Stages 3 and 4 were however, characterized by the expression of genes with important implications in early T-cell development such as ikaros (*IKZF1*), *CCL25α, MHCIIβ*, and *CD74α. IKZF1* is required for lymphocyte development in mice (54) and zebrafish (55, 56). CCL25α is a chemokine expressed in the thymic epithelium that attracts dendritic cells, thymocytes, and macrophages in mammals (22, 57), and seems responsible for thymus homing in zebrafish (58). The expression of both *CCL25α* and *IKZF1* in ballan wrasse coincides with the colonization of the thymus by precursors of lymphoid cells and precede the expression of the recombination activating gene-1 (*RAG1*) as it was described in zebrafish (56). The characterization of the *MHCII-β* chain in several teleosts allowed researchers to study the distribution of the main TCR-αβ T-cell populations within the thymus. For example, Atlantic salmon (32) and rainbow trout (33) presented abundant MHCII^+^ cells in the medulla compared to the outer region of the thymus suggesting that MHCII^+^ cells are involved in positive selection of developing T-cells in a similar fashion as in higher vertebrates (30, 32, 59). In line with this, Picchietti et al. (31) also demonstrated the higher abundance of MHCII^+^ cells in the medulla compared to the cortex in sea bass larvae. Interestingly, the authors observed an increase of *MHCII-β* transcripts at the same time as lymphoid precursors colonized the primordial thymus that was still devoid of cortex and medulla regions. Similarly, transcripts of wrasse *MHCII-β* and the *CD74α* gene, which codes for proteins involved in the formation of MHCII peptide complexes, increased extensively at the end of stage 4, shortly after thymocytes were observed in the thymus and before the organ became more prominent. Taking all together, the results indicate that the migration of lymphoid cell precursors and the creation of the optimal thymic environment for T-cell development starts at stage 3 and continues during stage 4, prior to the initiation of T-cell maturation. *MHCII-α* transcripts were present at developmental stage 2 and onwards, probably corresponding to populations of innate-like leukocytes.

A morphological change of the thymus occurs at the same time (stage 5) as transcripts of T-cell specific markers (*RAG1, RAG2, LCK, ZAP70, CD3δ, CD3ζ, CD3ϵ, CD4-1, CD4-2, CD8β, TCRα, and TCRδ*) increased significantly evidencing the start-point of T-cell maturation. Therefore, it seems likely that the thymus contributes to the overall expression of these genes. Wrasse developmental stage 5 include larvae that vary in size with a standard lentgh between 13 and 19 mm accounting for slightly different developmental sub-stages. This is the reason why transcriptomic data at this stage do not provide the exact order of each T-cell marker during T-cell maturation. However, it is plausible to assume that *RAG1* initiates the rearrangment of the *TCR* genes followed by the upregulation of other T-cell markers such as *LCK, ZAP70, CD3, TCRα* and *TCRδ* as it happens in other teleosts (13). Interestingly, *TCRδ* transcripts decreased whereas *TCRα* increased during stage 6, leading to a higher level of *TCR*-*αβ* transcripts compared to *TCR-γδ* transcripts in juveniles of ballan wrasse as reported in mammals (11). The larvae period from stages 4 to 5 is also when growth increases and the volume of the digestive organs such as gut, liver and pancreas increase dramatically (2).

The classical zonation of the thymus into medulla (inner zone) and cortex (outer zone) seem to vary within species. Several species such as carp (23), zebrafish, rainbow trout, sea bass, halibut, and turbot (*Scophthalmus maximus*), show distinction between zones as reviewed in Barraza et al. (13), and in larvae of rice-field eel (*Monopterus albus*) (60), whereas the cortico-medullary boundary in Atlantic salmon thymus still remains unclear (46) and contradictory results were published in flounder (*Paralichthys olivaceus*) (52). The present work shows a clear zonation into cortex and medulla in the developing thymus of ballan wrasse larvae from stage 5 and onwards, both regarding thymic morphology and gene expression patterns. The cortical region was densely packed with thymocytes, while the emergent medullary region had an increasing number of cells with a smaller nuclear to cytoplasm ratio. Moreover, RAG*1^+^* cells were restricted to the outer cortex area, whereas the medulla appeared almost *RAG1^-^* in all investigated larvae and juveniles. *CD4-1^+^* and *CD8β^+^* cell zones within the thymus also evidence a clear demarcation between cortex and medulla as seen with *RAG1*, alike previously reports in seabass, ginbuna carp, rainbow trout and Atlantic halibut (24, 26–29). The detection of *RAG1^+^* thymocytes in the cortex is likely to correspond to both DN (CD4^-^ CD8^-^) and DP (CD4^+^ CD8^+^) thymocytes as reported in mammals and other teleosts (16, 17, 27). Not surprisingly, there were fewer cells in the medulla compared to the cortex that were *CD4-1^+^* or *CD8β^+^*, as only a limited number of thymocytes survive both positive and negative selection processes, and can leave the thymus migrating towards secondary lymphoid organs. The C-C chemokine receptor 9β (CCR9β) has been suggested as a potential marker for thymocyte selection within the thymus of fish (61). Accordingly, ballan wrasse *CCR9β* transcripts were upregulated at stage 5 coinciding with the start-point of T-cell maturation where these two selection processes are needed.

The increased expression and localization pattern of the different T-cell marker genes within the thymus of wrasse larvae implies that the thymus becomes lymphoid at larval stage 5. Moreover, the detection of *CD3* ^+^ cells in the head kidney at stage 5, indicates that mature T-cells have migrated out from the thymus at this timepoint, supported by the identification of *CD4-1* and *CD8* positive cells in the head kidney at stage 6. Interestingly, more *CD4-1^+^* cells were found in the thymus of juveniles compared to *CD8β^+^* cells, and transcripts of *CD4-1* were more abundant compared to *CD8β* during larvae ontogeny. This higher abundance of *CD4-1^+^* cells were also seen in head kidney, suggesting a higher production of mature helper T-cells compared to cytotoxic T-cells in developing ballan wrasse larvae and juveniles. In the present study we also investigated the distribution of helper T-cells (*CD4-1^+^ CD3ϵ^+^*) and cytotoxic T-cells (*CD8β^+^ CD3ϵ^+^*) in mucosal organs of developing larvae. Helper T-cells (*CD4-1^+^ CD3ϵ^+^*) were observed in the gut, gill, and pharynx of wrasse juveniles whereas cytotoxic T-cells were not found in any mucosal tissue at any investigated stage. However, we cannot exclude the possibility that cytotoxic T-cells are present in gut and gill of wrasse larvae. Although the method allowed for identification of *CD8β^+^* cells within the thymus, this organ contains an extraordinarily high number of *CD8β^+^* cells which is different to mucosal organs where much fewer T-cells are expected. Therefore, the sensitivity of the method might not have been high enough to allow detection of *CD8β^+^* T-cells in mucosal organs. Nevertheless, in accordance with these observations, our transcriptome data showed a higher number of *CD4-1* transcripts compared to *CD8β*. CD4 is also expressed by few sub-populations of dendritic cells and macrophages in teleosts (29, 62) and therefore, it is itself not an exclusively marker of helper T-cells. However, the fact that transcripts of both *CD4-1* and *CD8β* were upregulated at the same time during larvae development, and that *CD4-1* was not detected at an earlier stage as part of possible innate-like leukocytes, suggest that T-cells contribute to the overall expression of these genes at least during the investigated larvae stages. Therefore, the higher abundance of *CD4-1* transcripts supports the higher presence of helper T-cells in the thymus and mucosal organs of wrasse juveniles compared to cytotoxic T-cells indicating an important role of helper T-cells during early larval stages. This is in agreement with the fact that adaptive immunity needs to be stimulated by helper-T cells (62). Furthermore, ballan wrasse is a stomach-less species with a remarkably elevated immune activity in the hindgut (14, 15) that has been proposed to strategically compensate for the lack of stomach (14). One possibility is that the plausible high concentration of secreted immunoglobulins in the gut efficiently act as first line of defense against pathogens that are not inactivated by the acidic environment in the stomach. Abundant intraepithelial IgM^+^ cells were observed within the gut of adult wrasse together with an extraordinary high amount of IgM in plasma compared to other teleosts (15). We hypothesize that helper T-cells are especially important to boost B-cell activation and antibody production in gut and other mucosal organs in early stages of wrasse larvae when they are most susceptible to diseases and pathogens.

RAG1 and RAG2 are crucial for T- and B-cell receptor rearrangment in developing lymphocytes, processes that are described in primary lymphoid organs, the thymus and head kidney in teleosts (18, 19, 48, 63). However, intraepithelial lymphocytes in the gut of humans (64) and mice (65, 66) are RAG^+^ suggesting the presence of maturing B- and T-cells within the intestine. Similarly, T-cells isolated from the gut of adult European sea bass express *RAG1* (67). In zebrafish, *RAG1^+^* cells were reported in the gut of adult individuals (68) and a few putative T-cells expressing *RAG1* were reported in the gut of carp at 1 week post fertilization (69). Furthermore, the same authors used a monoclonal antibody for putative intraepithelial T-cells and positive cells were found within the gut prior to the identification of thymocytes in the thymus. Even though the gut is not considered a lymphoyd tissue but rather a tissue containing abundant lymphoid cells in higher vertebrates, Scapigliati et al. (70) suggested the gut of adult fish to be a lymphoid tissue that has retained a primordial lymphopoietic function throughout evolution. The expression of *RAG1* in mucosal organs was also investigated in ballan wrasse larvae (data not shown). There was a weak expression of *RAG1* in gill and gut that did not seem to correspond to leukocytes. Noticeable, positive signals were found in non-lymphoid tissue such as brain and eye which are typically used as internal negative controls for the expression of these immune genes. Furthermore, *RAG1* transcripts in the gut of adult wrasse appear to be absent (3). Althogether, the results indicate lack of *RAG1* expression in mucosal organs and no evidence for extrathymic development of T cells in ballan wrasse.

It is well established that the innate and adaptive immune systems are extensively related with no clear border between them. For instance, T-cells that are classically described as adaptive immune cells may have adaptive (TCR based) functions as well as innate (cytokine based) functions making interactions between innate and adaptive systems crucial for a successful immune response. When we keep an animal in captivity we have the obligation to meet the dietary requirements for the animal at all developmental stages to ensure healthy and robust fish. This is enherentley difficult, especially in larvae where requirements are poorly understood (71). Feed and feed additives can modulate the immune system as indicated in many studies (37, 72, 73).

The total amounts of PUFAs (n-3 and n-6) as well as the dietary DHA/EPA (n-3) ratios are important for growth, reproduction, and survival (74, 75). Modulation of the dietary DHA/EPA ratio and the level of ARA has been reported to affect certain immune responses, however, mostly related to the innate immune system (76–78). In Atlantic halibut larvae, it was suggested that the low level of dietary n-3-HUFA, especially DHA, could be the direct cause of several developmental errors (79). Later, Øvergård et al. (24) analysed T-cell development of halibut larvae and suggested that dietary fatty acid composition of the live feed seemed to modulate the expression of several T-cell marker genes during larvae ontogeny. In the present work, total levels of PUFAs and DHA were highest in rotifers and similar in artemia and barnacles, due to good enrichment practices. However, barnacles showed higher EPA and lower ARA levels compared to rotifers and artemia. EPA and ARA are precursors of eicosanoids and can be anti- and pro-inflammatory, respectively. Thus, the ratio of dietary EPA/ARA determines eicosanoid production and health status accordingly (80). As a general rule, derivates from EPA have been considered anti-inflammatory, while ARA derivates are considered pro-inflammatory (81). Although there may be evidence that this is different or varies in teleosts (82), the abundant levels of EPA found in barnacle nauplii used in the present work could trigger the production of anti-inflammatory eicosanoids as well as enhancing the total amount of PUFA boosting earlier development of adaptive immunity.

Rotifers and artemia were higher in all protein bound amino acids, but not the FAAs proline and taurine. Proline was the most abundant FAA in the barnacle diet which is similar to that reported in wild copepods and zooplankton (83). Taurine is involved in many biological functions and it is important for a successful development of marine larvae (84–86). Its deficiency can cause oxidative stress and lipid accumulation among others (85, 87, 88). Northern rock sole (*Lepidopsetta polyxystra*) larvae performed better when fed with rotifers enriched with taurine (84). Authors observed that taurine supplementation yielded larvae with higher dry weight but not higher standard length, as observed in the present work in ballan wrasse. Taurine was higher in small barnacle compared to rotifers but higher in artemia compared to large barnacle, in contrast to copepod nauplia that are well characterized for having high levels of taurine compared to rotifers and artemia (86). Taking into account the beneficial role of taurine for larvae development (84, 86), we speculate that the relatively low levels of taurine present in barnacles might cause the lack of a clear positive effect of the barnacle diet on the performance of wrasse larvae.

Extensive work on the requirements for minerals and vitamins for marine larvae has been reported (71, 86). Mæhre et al. (86) recommended that the composition of zooplankton, which is the natural prey of most marine larvae should be used as reference. Vitamins have broad implications such as antioxidants and immune modulators, and deficiencies can cause large losses in marine larvae production as reviewed in (89). Enriched rotifers and artemia showed higher amount of all investigated vitamins compared to barnacles in the present work. Vitamin A was under the detection limit which is comon in zooplankton although ballan wrasse can probably convert both astaxanthin and canthaxanthin to vitamin A as it happens in other fish species (90, 91). The astaxanthin levels in the barnacle diets were similar to enriched rotifers and artemia whereas canthaxanthin was only present in barancles at low levels. This indicates that a diet composed solely of barnacle nauplii might contain lower levels of natural carotenoids or at least, a different profile than wild zooplankton (83, 92). All the vitamins in barnacles were present at a much lower levels than in wild harvested copepods (71, 83).

Overall, iodine was the only mineral that was higher expressed in both small and large barnacle compared to rotifers and artemia. Iodine supplementation in larvae has been reported to improve the thyroid hormone status in halibut and Senegalese sole larvae (*Solea senegalensis*) (93, 94). Furthermore, thyroid hormones are modulators of the innate immune system and have implications on cells that are important for mounting an adaptive response (95). More differences in the mineral content were observed when comparing the start-feed diets based on the timeline when they were administrated to the tanks; small barnacle were significantly higher in V, Mn, Co, Zn, As, Se, and Ca compared to rotifers. Rotifers and small barnacle nauplii were the first foods given to larvae. Ma, Co and Se are important co-factors in antioxidant enzymes and therefore, important to protect against lipid oxidation (71). Optimal levels of selenium enhance the innate response and deficiency decreases the number of B-cells in humans (36). Zinc and selenium are naturally more abundant in copepods compared to enriched rotifers (71, 86) which is in accordance with our results. Interestengly, zinc deficiency seems to affect primarily T-cells, leading to apoptosis of double positives thymocytes in the cortex and thymic atrophy (34, 36). Artemia and large barnacle nauplii were introduced at 19 dph and 15 dph correspondingly and had similar mineral profiles.

Nutritional requirements for larvae are difficult to investigate and studies adressing this matter are rather scarce (83, 89, 96). In this study, barnacle-fed larvae showed that important genes related to T-cell development were upregulated earlier and the size of the thymus was larger. A clear correlation between the size of the thymus and its capacity to produce mature T-cells is lacking in teleosts (13, 23, 97). Although we did not address the abundance of lymphocytes in the thymus of investigated larvae, it is likely that the experimental barnacle feed was favourable regarding T-cell development and possibly reflecting a healthier larvae production. The experimental diet composed solely of barnacle nauplii seems to be somewhat low in important nutrients characteristic of a zooplankton diet such as taurin, vitamins and carotens. On the other hand, the barnacle nauplii diet was higher in EPA with a high n-3/n-6 ratio, it contained higher levels of iodine as well as microminerals such as Mn, Co, Se, and zinc. These nutrtional traits might be directly related to the earlier onset of adaptive immunity in ballan wrasse larvae.

## 5 Conclusion

Similar to other teleosts, the thymus of ballan wrasse becomes lymphoid at stage 5 of larvae development. At this stage, there is a clear distinction between the cortex, where TCR rearrangement takes place and thymocytes are *RAG1^+^*, and the medulla, which is involved in negative selection processes where most T-cells are *RAG1^-^*. Although it seems that a cortico-medullary division is present in most teleosts being a potential common feature of bony fish, there is not yet consensus on its organization. The localization of *RAG* is a key tool for thymus zonation (98) and should be used for elucidating existing disagreements. Wrasse larvae at stage 6 and juveniles possess helper T-cells in mucosal organs which might be crucial to activate antibody-secreting B-cells and recruit other leukocytes to the gut of this a-gastric species. Although results indicate that immunological competence is present at least, to some extent in juveniles of ballan wrasse, the study of B-cell development and the timing of appearance of IgM^+^cells able to secrete IgM is needed for establishment of effective prophylactic measures. Interestingly, a start-feed diet composed of barancle nauplii seems to trigger an earlier onset of adaptive immunity in ballan wrasse larvae.

## Data availability statement

The datasets presented in this study can be found in online repositories. The names of the repository/repositories and accession number(s) can be found below: https://www.ncbi.nlm.nih.gov/, GSE200208.

## Ethics statement

The animal study was reviewed and approved by The Directorate of Fisheries of Norway (permission nr. VL-AV-0011 given to the Institute of Marine Research station at Austevoll (location nr. 16195)). The experiment and sampling followed the Norwegian animal welfare act guidelines, in accordance with the Animal Welfare Act of 20th December 1974, amended 19th June 2009. The facility has a general permission to conduct experiments involving all developmental stages of fish (code 93) provided by the Norwegian Animal Research Authority (FDU, www.fdu.no).

## Author contributions

ØS, IH, and AE designed the experiments. AE and RB participated in carrying out the experiment. AE, ØS, KL, and IH: analyses. AE wrote the manuscript. AE, IH, A-CØ, KL, and ØS: editing. All authors contributed to the article and approved the submitted version.
