# Supplementary material for: The thymus and T-cell ontogeny in ballan wrasse (Labrus bergylta) is nutritionally modelled
Source: Front Immunol. 2023 May 1;14:1166785. doi: 10.3389/fimmu.2023.1166785 (PMC10183603; doi:10.3389/fimmu.2023.1166785)
Supplement: Supplementary file 1 [file DataSheet_1.docx]

Supplementary Material

The thymus and T-cell ontogeny in ballan wrasse (*Labrus bergylta*) is nutritionally modelled

**Angela Etayo^1,2^*†, Kai K. Lie^1^, Reidun M. Bjelland^3^, Ivar Hordvik^2^, Aina-Cathrine Øvergård^2^ and Øystein Sæle^1^**

^1^ Institute of Marine Research, Bergen, Norway

^2^ Fish Health group, Department of Biological sciences, University of Bergen, Norway

^3^ Institute of Marine Research, Austevoll Research Station, 5392 Storebø, Norway

*** Correspondence:**

Angela Etayo

Email: [angela.etayo@uib.no](mailto:angela.etayo@uib.no)

# Supplementary Data

## Supplementary Data 1. Cultured conditions of rotifers, artemia and barnacles used in the experiment.

A deep inlet of full seawater from the proximity of the facility in Austevoll was used for rearing of rotifers, artemia and barnacles. The same water was used for larvae production. The culture conditions of live prey feed were as following:

- Small and large barnacle nauplii (planktonic diet) were provided by @planktonic as cryoPlankton shipped in cytogenetic dewars (liquid nitrogen at -196 ºC). Preparing the live feed was done in a few simple steps that included thawing, rinsing, and revitalizing the nauplii until their normal swimming activity was resumed. Thawing and rinsing was done for few minutes with continuous stirring followed by revitalization of the nauplii with > 50 % oxygen saturation overnight at <5 ºC (Revitalized nauplii have a maximum of 36 hours storage). At this stage the nauplii were ready to be fed to the fish larvae and were brought to the tank facility where they were bumped into the tanks.
- The rotifer strain at the Austevoll Research Station is a uniform culture of *Branchonus plicatilis* "cayman". Rotifers are fed on algae *Nannochloropsis* and *Tetraselmis* that is shipped as a frozen product from the Norwegian supplier Microalgae AS, which is based at Vigra. Dry yeast is also used as growth feed (egg development) in rotary culture. Multigrain is used as enrichment (<https://www.biomar.com/en/larviva/hatchery-for-fish/emea/>). The amount of Multigrain was 0.15 g per million rotifers. The production tanks were kept at 22.5 ± 1ºC with an oxygen saturation of 80%. Thoroughly washed rotifers were transferred to the tank and fed every hour by automatic feeding. The rotifers in the production tank were transferred to the enrichment tank through a washing station where rotifers were washed before enrichment. The enrichment tanks have a density of 1500 ± 500 rotifers/ml = 1.5 ± 05 million rotifers/liter, and a temperature of 22.5 ± 0.5 ºC with an oxygen saturation of 100% was kept constant. A total of 0.15 g Multigrain per million rotifers is added to the tanks. Quality controls of enriched rotifers were made to check density, vitality and contamination before being administrated to the larvae. The vitality of the rotaries is assessed based on swimming activity.
- Artemia cyst (EG Artemia Cysts) are bought from INVE Aquaculture (<https://www.inveaquaculture.com/product/artemia-eg-gsl-inve-blue/> ). The cyst density in the hatching tank did not exceed 2.5g cysts/ liter of seawater. The hatching tank was kept at 28 ºC with an oxygen saturation of 100-200 %, continuous light and salinity level of 33-35%. The cysts hatched after 24-26 hours, then the artemia was washed at least for 10 min before being transferred to the enrichment tanks. Enrichment tanks are kept at 26 ºC, with an oxygen saturation of 100-200 % and a salinity level of 33-35 %. The artemia nauplii were transferred to the enrichment tank in the afternoon where 1L of the multigrain was delivered automatically 4 times per day, the last feeding the following day at 6 am. The artemia was washed first in temperate seawater for 3 min, posteriorly with freshwater for 10 min or until the salinity was <0,3 and a last wash in cold seawater until the salinity was >33%. The artemia was then quality checked and concentrated into the feeding tank. Vitality, and the degree of gut filling with multigrain was checked at this point and administered to the larvae.

Live prey (rotifers, artemia, and barnacle) was administrated to the corresponding tanks by slowly pumping 3 times per day (morning, afternoon, and evening). The evening meal was given at a slower speed so that the larvae had food available overnight until the next morning feeding occurred.

# Supplementary Figures and Tables

## Supplementary Figures


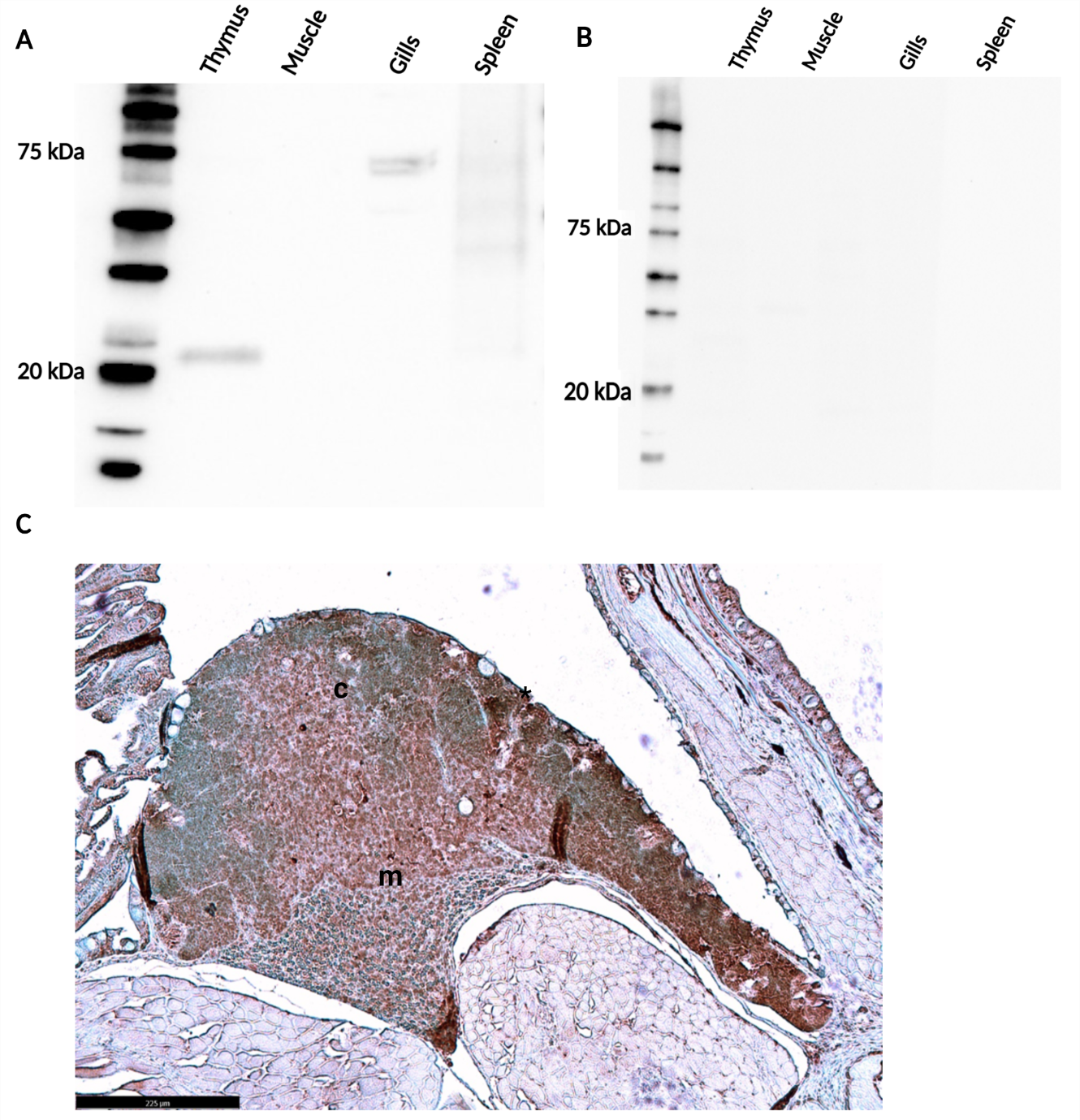


**Supplementary Figure 1.** **(A)** Western blot of protein extracts from ballan wrasse tissues incubated with an affinity purified rabbit polyclonal anti-CD3ε antibody. The expected molecular mass of wrasse CD3e is 19,6 kDa. A positive band at the expected molecular weight was observed in thymus while being absent in the rest of the investigated tissues. **(B)** Immunoblot omitting primary antibody. **(C)** Cross-sections of head from ballan wrasse larva at stage 6 showed CD3ε^+^ cells expressed in the thymus visualized with brown staining. Epithelial staining is due to a cross reaction with a 75 kDa protein. Background stain: methyl green. Antibody dilution 1:100. m: medulla. c: cortex. Asterisks indicate gill cavity. Scale bar = 225 μm.

## Supplementary Tables

**Supplementary Table 1.** References of the methodology used for nutrient analysis.

| Analyses | Principle | Reference |
| --- | --- | --- |
| Vitamin C | HPLC | [(Mæland and Waagbø, 1998)](https://www.sciencedirect.com/science/article/pii/S1095643398101253) |
| Biotin | Microbiological fertilization | ([Mæland et al., 2000](https://link.springer.com/article/10.1007/s002270000280)) |
| Folate | Microbiological fertilization | [(Mæland et al., 2000)](https://link.springer.com/article/10.1007/s002270000280) |
| Niacin | Microbiological fertilization | [(Mæland et al., 2000)](https://link.springer.com/article/10.1007/s002270000280) |
| Pantoten | Microbiological fertilization | [(Mæland et al., 2000)](https://link.springer.com/article/10.1007/s002270000280) |
| Vitamin B6 | HPLC | [(CEN, 2005)](https://standards.iteh.ai/catalog/standards/cen/68971dba-e41e-44f7-b544-b6c8e5776de4/en-14663-2005) |
| Thiamine | HPLC | (CEN, 2003a) |
| Riboflavin | HPLC | [(CEN, 2003b)](https://standards.iteh.ai/catalog/standards/cen/0472ce56-5fec-49bb-ac22-9ab85768ef94/en-14152-2014) |
| Cobalamin | Microbiological fertilization | ([Mæland et al., 2000)](https://link.springer.com/article/10.1007/s002270000280) |
| Vitamin A | HPLC | [(Moren et al., 2004)](https://www.sciencedirect.com/science/article/pii/S0044848604000523) |
| Vitamin D | HPLC | (CEN, 1999) |
| Vitamin E | HPLC | [(Hamre et al., 2010)](https://onlinelibrary.wiley.com/doi/full/10.1111/j.1365-2109.2009.02375.x) |
| Vitamin K | HPLC | (CEN, 2003c) |
| Astaxanthin | HPLC | ([CEN,2011](https://standards.iteh.ai/catalog/standards/cen/cf07d5a4-d693-40dc-b211-a8bab85a75fa/cen-ts-16233-1-2011)) |
| Canthaxanthin | HPLC | [(CEN,2011)](https://standards.iteh.ai/catalog/standards/cen/cf07d5a4-d693-40dc-b211-a8bab85a75fa/cen-ts-16233-1-2011) |
| Minerals | ICP-MS | [(Long and Martin 1990)](https://www.osti.gov/biblio/6681306) |
| Iodine | ICP-MS | [(Julshamn et al., 2004)](https://www.tandfonline.com/doi/abs/10.1080/02652030310001639512) |
| HAA (hydrolysed amino acids) | HPLC-UV | [Waters, AccQ-TagTM Method. 715001320](https://www.waters.com/webassets/cms/support/docs/71500129702rb.pdf) |
| Fatty Acid | GC-FID | [(Torstensen, Espe et al. 2011)](https://www.cambridge.org/core/journals/british-journal-of-nutrition/article/dietary-plant-proteins-and-vegetable-oil-blends-increase-adiposity-and-plasma-lipids-in-atlantic-salmon-salmo-salar-l/C7111567384E90A6819B3C1212875B5A) |
| Crude Protein | Combustion | [(Simonne et al., 1997)](https://onlinelibrary.wiley.com/doi/abs/10.1002/(SICI)1097-0010(199701)73:1%3C39::AID-JSFA717%3E3.0.CO;2-4) |
| Crude Lipid-ethyl acetate | Isopropanol extraction | [NS 9402:1994](https://www.standard.no/en/PDF/FileDownload/?redir=true&filetype=Pdf&preview=true&item=135397&category=5) |
| Ash | Combustion | [(AOAC, 1942)](https://academic.oup.com/jaoac/article/95/5/1392/5655282) |
| Dry weight | Gravimetric after freeze drying | [(Hamre et al., 2006)](https://onlinelibrary.wiley.com/doi/10.1111/j.1365-2095.2006.00377.x) |

**Supplementary Table 2.** Nutrient analyses of the start-feed diets

|  | Rotifers | Artemia | Small barnacle | | Large barnacle |
| --- | --- | --- | --- | --- | --- |
| **Proximate composition (g/ 100g DW)** | | | |  |  |
| Protein | 51±0^a^ | 36±9^b^ | 42±17^abc^ | | 52±22^ac^ |
| Lipid | 16±4 | 13±6 | 8±4 | | 9±2 |
| Ash | 36±11 | 23±1 | 30±18 | | 23±6 |

Analyzed dietary proximate in rotifers, Artemia, small barnacle (*Balanus crenatu*), and large barnacle (*Semibalanus balanoides).*

Values are relative to dry weight (DW) and are given as mean ± SD when value is >1. The number of replicates is 3 (N=3). Significances are indicated by letters.

|  | Rotifers | Artemia | Small barnacle | Large barnacle |
| --- | --- | --- | --- | --- |
| **Fatty acids (% of TFA)** | | | | |
| **ΣSFA** | **22±5** | **22** | **22** | **19** |
| 14:0 myristic acid | 2±1^a^ | 1^a^ | 6^b^ | 3±1^a^ |
| 16:0 palmitic acid | 18±4 | 14 | 13 | 13±1 |
| 18:0 Stearic acid | 2 | 5 | 2 | 2 |
| **ΣMUFA** | **9±1^a^** | **28±1^b^** | **25^b^** | **20±2^c^** |
| 16:1n-7 Palmitoleic acid | 3^a^ | 5^a^ | 11^b^ | 5±2^a^ |
| 18:1n-9 Oleic acid | 3^a^ | 13±1^b^ | 4^ac^ | 5±1^c^ |
| 18:1n-7 Vaccenic acid | 1^a^ | 8^b^ | 7^b^ | 6^b^ |
| **ΣPUFA** | **65±6^a^** | **45±1^b^** | **50^bc^** | **58±3^ac^** |
| **Σn-3** | **46±5^a^** | **31±1^b^** | **47±1^a^** | **55±3^a^** |
| 18:3n-3 α-Linolenic acid | 0^a^ | 9±2^b^ | 0^a^ | 1^a^ |
| 18:4n-3 Stearidonic acid | 0 | 1 | 2 | 2 |
| 20:5n-3 EPA | 7±1^a^ | 9^a^ | 28^b^ | 32±4^b^ |
| 22:6n-3 DHA | 34±6^a^ | 11±2^b^ | 15^b^ | 19±2^b^ |
| **DHA/EPA** | 5 | 1 | 1 | 1 |
| **Σn-6** | **19±1^a^** | **14^b^** | **2^c^** | **3±1^c^** |
| 18:2n-6 LA | 4±1^a^ | 6^b^ | 1^c^ | 1^c^ |
| 20:4n-6 ARA | 2^a^ | 3^b^ | 1^c^ | 1^c^ |
| **n-3/n-6** | **2^a^** | **2^a^** | **26±2^b^** | **21±7^b^** |
| **TFA (µg/Kg DW)** | **4593±934** | **1323±144** | **631±289** | **777±252** |

Analyzed fatty acid composition and total fatty acids (TFA) in rotifers, Artemia, small barnacle (*Balanus crenatu*), and large barnacle (*Semibalanus balanoides).*

Values are relative to dry weight (DW) and are given as mean ± SD when value is >1. The number of replicates is 3 (N=3). Values indicated as trace amounts (tr) correspond to values that were detected but are below quantification limits of the analytical method. Significances are indicated by letters. TFA - total fatty acids; SFA – saturated fatty acids; MUFA - monounsaturated fatty acids; LA – linoleic acid, ARA-arachidonic acid, EPA - eicosapentaenoic acid; DHA - docosahexaenoic acid; PUFA - polyunsaturated fatty acids.

Analyzed minerals in rotifers, Artemia, small barnacle (*Balanus crenatu*), and large barnacle (*Semibalanus balanoides).*

|  | Rotifers | Artemia | Small barnacle | Large barnacle |
| --- | --- | --- | --- | --- |
| **Micro-mineral composition (µg kg^-1^ DW)** | | | |  |
| V | 5±1^a^ | 34±47^ab^ | 88±5^b^ | 26±12^ab^ |
| Cr | 4±2 | 32±30 ^*^ | 49±6 | 87±102 |
| Mn | 98±29^a^ | 313±429^a^ | 1170±142^b^ | 159±84^ac^ |
| Fe | 2020±586 | 11496±15911 | 26590±1421 | 15313±9672 |
| Co | 2±1^a^ | 7±9^ab^ | 16±1^b^ | 6±4^ab^ |
| Ni | 3* | 90** | 65±8 | 45±13 |
| Cu | 122±39 | 158±76 | 136±4 | 181±96 |
| Zn | 203±85^a^ | 2220±2536^ab^ | 4343±667^b^ | 5825±843^b^ |
| As | 1^a^ | 84±61^ab^ | 165±52^bc^ | 295±53^c^ |
| Se | 3±1^a^ | 10±7^ab^ | 19±3^b^ | 15±2^b^ |
| Mo | 5±1* | 15** | 10 | 8±1 |
| Ag | tr | 1* | 1* | 0.5* |
| Cd | 1 | 8** | 6±1 | 4±2 |
| Hg | tr | tr | 0.3 | 0.2 |
| Pb | 6±2 | 14±14 | 19±4 | 5±4 |
| Jod | 58±26^a^ | 244±300^a^ | 2819±523^b^ | 736±154^c^ |
| **Macro-mineral composition (mg kg^-1^ DW)** | | | | |
| Ca | 39^a^ | 83±36^ab^ | 460±225^b^ | 284±139^ab^ |
| Na | 584±105^a^ | 1721±451^b^ | 753±322^a^ | 673±234^a^ |
| K | 109±6 | 111±17 | 73±7 | 80±26 |
| Mg | 66±10^a^ | 207±35^b^ | 117±32^a^ | 99±32^a^ |
| P | 115±16 | 66±16 | 94±23 | 115±13 |

Values are relative to dry weight (DW) and are given as mean ± SD when value is >1. The number of replicates is 3 (N=3) unless otherwise specified by *. Values indicated as trace amounts (tr) correspond to values that were detected but are below quantification limits of the analytical method. ANOVA test was applied only when N=3 and values are numeric (nutrients with trace elements in at least one of the diets, were not legit for statistics). Significances are indicated by letters.

^*^ N=2. One out of the three samples analyzed were found in trace amounts and the expressed value corresponds to two replicates.

^**^ N=1, no SD. Two out of the three samples analyzed were found in trace amounts and the expressed value corresponds to only one replicate.

Analyzed vitamins and pigments in rotifers, artemia, small barnacle (*Balanus crenatu*), and large barnacle (*Semibalanus balanoides).*

|  | Rotifers | Artemia | Small barnacle | Large barnacle |
| --- | --- | --- | --- | --- |
| **Water soluble vitamins (µg kg^-1^ DW)** | | | |  |
| Vitamin C Ascorbic acid | 23363±3575^a^ | 7275±1138^b^ | 512±373^b^ | 5209±6130^bc^ |
| Vitamin B1 Thiamine | 1374±934 | 76±12 | 36±16 | 35±4 |
| Vitamin B2 Riboflavin | 479±228 | 312±23 | 322±123 | 471±113 |
| Vitamin B3 Niacin | 4111±1820^a^ | 1413±161^ab^ | 310±129^b^ | 660±145^b^ |
| Vitamin B6 Pyridoxine | 412±224 | 230±90 | 79±45 | 76±6 |
| Vitamin B7 Biotin | 53±28^a^ | 36±2^ab^ | 4±1^b^ | 7±1^b^ |
| Vitamin B9 Folate | 141±15^a^ | 93±1^b^ | 22±24^c^ | 9±8^c^ |
| Pantoten | 2622±1041^a^ | 904±24^b^ | 343±190^b^ | 658±213^bc^ |
| Vitamin B12 Cobalamin | 59±33^a^ | 53±2^a^ | 4±1^b^ | 6±3^b^ |
| **Lipid soluble vitamins (µg kg^-1^ DW)** | | | |  |
| Vitamin A1 | tr | tr | tr | tr |
| Vitamin A2 | tr | tr | tr | tr |
| Vitamin D3 Cholecalcipherol | 4±3 | 2±1 | tr | tr |
| Vitamin E ^1^ | 24536±16785^a^ | 5360±1855^ab^ | 889±393^b^ | 884±173^ab^ |
| Vitamin K ^2^ | 105±7^a^ | 13±6^b^ | 8±2^b^ | 3±1^b^ |
| **Pigments (µg kg^-1^ DW)** | | | |  |
| Astaxanthin | 1374±912 ^a^ | 41±2 ^ab^ | 28±20^b^ | 60± 10 ^ab^ |
| Canthaxanthin | tr | tr | 4** | 7** |

Values are relative to dry weight (DW) and are given as mean ± SD when value is >1. The number of replicates is 3 (N=3) unless otherwise specified by *. Values indicated as trace amounts (tr) correspond to values that were detected but are below quantification limits of the analytical method. ANOVA test was applied only when N=3 and values are numeric (nutrients with trace elements in at least one of the diets, were not legit for statistics). Significances are indicated by letters.

^1^ The value is the sum of tokoferol-α/β/γ/δ, and tocotrienol- α/β/γ/δ

^2^ The value is the sum of vitamin k1, ß,Y-Dihydro vitamin K1, and the different Vitamin K2 (MK4, MK5, MK6, MK7,MK8, MK9, and MK10).

^**^ N=1, no SD. Two out of the three samples analyzed were found in trace amounts and the expressed value corresponds to only one replicate.

Analyzed protein-bound amino acids (PAA) and free amino acids (FAA) in rotifers, artemia, small barnacle (*Balanus crenatu*), and large barnacle (*Semibalanus balanoides).*

|  | Rotifers | Artemia | Small barnacle | Large barnacle |
| --- | --- | --- | --- | --- |
| **Protein-bound amino acids (PAA)** | | | | |
| **Essential amino acids (µg/Kg dw)** | | | | |
| Histidine | 207** | 76** | 63±28 | 62±2 |
| Arginine | 695±92 | 255±1 | 200±88 | 203±7 |
| Threonine | 570±76 | 197±1 | 150±67 | 146±5 |
| Lysine | 1007±183 | 378±2 | 214±102 | 218±18 |
| Methionine | 266±34 | 92 | 65±29 | 69±4 |
| Valine | 683±105 | 225 | 152±69 | 155±8 |
| Isoleucine | 653±103 | 206±1 | 110±50 | 108±6 |
| Leucine | 1018±152 | 315±1 | 199±89 | 203±12 |
| Phenylalanine | 612±73 | 175±1 | 118±52 | 112±4 |
| **Non-essential amino acids (µg/Kg dw)** | | | | |
| Serine | 681±91 | 205±1 | 164±72 | 157±1 |
| Glycine | 549±77 | 203±1 | 226±99 | 198±2 |
| Aspartic acid | 1461±232 | 440±5 | 288±133 | 272±16 |
| Glutamic acid | 1845±281 | 598±10 | 370±170 | 329±17 |
| Alanine | 611±86 | 270±3 | 186±88 | 190±9 |
| Hydroxyproline | tr | tr | tr | tr |
| Proline | 676±92 | 195±1 | 188±86 | 226±10 |
| Tyrosine | 490±54 | 155 | 121±55 | 121±5 |
| **Free amino acids (FAA)** | | | | |
| **Essential amino acids (µg/Kg dw)** | | | | |
| Histidine | 28±3 | 20±1 | 4±2 | 4 |
| Arginine | 93±21 | 88±1 | 23±10 | 27±2 |
| Threonine | 50±9 | 34±2 | 4±2 | 5 |
| Lysine | 108±18 | 74 | 10±4 | 13 |
| Methionine | 34±6 | 28±1 | 4±2 | 5±1 |
| Valine | 55±8 | 46±2 | 5±2 | 6 |
| Isoleucine | 57±10 | 44±2 | 3±1 | 3 |
| Leucine | 96±18 | 64±1 | 7±3 | 6 |
| Phenylalanine | 66±13 | 43±1 | 4±2 | 5 |
| Tryptophan | 10±2 | 11±1 | 3±1 | 3 |
| **Non-essential amino acids (µg/Kg dw)** | | | | |
| Serine | 76±15 | 41±2 | 6±3 | 6 |
| Glycine | 37±6 | 25±1 | 19±8 | 19±1 |
| Aspartic acid | 54±9 | 25±1 | 3±1 | 2 |
| Glutamic acid | 149±24 | 74±4 | 15±7 | 16±1 |
| Alanine | 80±10 | 64 | 21±9 | 24±1 |
| Proline | 52±7 | 42±2 | 35±15 | 67±4 |
| Tyrosine | 61±10 | 47±1 | 10±4 | 9±1 |
| **Glutamine** | 66±13 | 47±2 | 5±2 | 3 |
| **Gamma-amino butyric acid** | 0 | 0 | 1 | 1 |
| **Asparagine** | 61±10 | 38±2 | 3±1 | 3 |
| **Taurine** | 3±1 | 35 | 17±7 | 25±2 |

Values are relative to dry weight (DW) and are given as mean ± SD when value is >1. The number of replicates is 2 (N=2) unless otherwise specified by *. Values indicated as trace amounts (tr) correspond to values that were detected but are below quantification limits of the analytical method. Significances were not applied as N was considered too low (N=2).

^**^ N=1, no SD. Two out of the three samples analyzed were found in trace amounts and the expressed value corresponds to only one replicate.
